# Curvularin Isolated From *Phoma macrostoma* Is an Antagonist of RhlR Quorum Sensing in *Pseudomonas aeruginosa*

**DOI:** 10.3389/fmicb.2022.913882

**Published:** 2022-07-12

**Authors:** Ha-Young Choi, Duc Dat Le, Won-Gon Kim

**Affiliations:** ^1^Infectious Disease Research Center, Korea Research Institute of Bioscience and Biotechnology, Daejeon, South Korea; ^2^Department of Bio-Molecular Science, KRIBB School of Bioscience, Korea University of Science and Technology (UST), Daejeon, South Korea

**Keywords:** anti-quorum sensing, curvularin, RhlR, antagonist, *Pseudomonas aeruginosa*

## Abstract

Quorum sensing (QS) is an attractive target for the treatment of multidrug-resistant *Pseudomonas aeruginosa*, against which new antibiotics are urgently needed. Because LasR is at the top of the QS hierarchy controlling Rhl and PQS systems, most QS inhibitors have been targeted to LasR. However, it has recently been reported that in clinical isolates of *P. aeruginosa*, LasR is frequently mutated and nonfunctional, and RhlR independently acts to produce virulent factors that maintain toxicity. Thus, for effective treatment of chronic cystic fibrosis infections, RhlR antagonists is needed to prevent the LasR-independent Rhl system, but RhlR antagonists have rarely been reported. In this study, we found that curvularin, an aromatic compound with a cyclized alkyl side chain isolated from *Phoma macrostoma*, at a low micromolar concentration of 1–30 μM potently and selectively inhibited pyocyanin and rhamnolipid production without affecting the cell viability of *P. aeruginosa*. Only high concentration (more over 100 μM) curvularin negligibly inhibited biofilm formation and elastase production, suggesting that curvularin at low concentrations selectively inhibits RhlR. The QS antagonism by curvularin was investigated in experiments using QS competition and signaling molecules assays with QS gene expression analysis, and the results showed that, indeed, at low concentrations, curvularin selectively antagonized RhlR; in contrast, it negligibly antagonized LasR only when applied at a high concentration. The exclusive RhlR antagonizing activity of curvularin at low concentrations was confirmed using QS mutants; specifically, curvularin at low concentrations inhibited pyocyanin and rhamnolipid production by selectively antagonizing N-butanoyl homoserine lactone (BHL)-activated RhlR. Moreover, by targeting RhlR, curvularin reduced the *in vivo* virulence of wild-type *P. aeruginosa* as well as *lasR* mutants in *Caenorhabditis elegans*. Overall, low-concentration curvularin is a pure RhlR antagonist in *P. aeruginosa*, and to the best of our knowledge, this is the first report describing an RhlR antagonist from natural resources. Hence, curvularin has great potential for the development of chronic *P. aeruginosa* infection therapeutics and for the study of RhlR function in the complex QS system.

## Introduction

*Pseudomonas aeruginosa* is a ubiquitous Gram-negative bacterium that causes many opportunistic and nosocomial infections. In particular, chronic lung infections by *P. aeruginosa* are the leading causes of mortality in cystic fibrosis (CF) patients (Finnan et al., 2004). The World Health Organization recently placed it in the 12 antibiotic-resistant “priority pathogens” (Donlan and Costerton, 2002; Davies, 2003; World Health Organization, 2017). *Pseudomonas aeruginosa* produces several virulence factors including elastase, rhamnolipid, and pyocyanin, and also forms thick biofilm in the CF lung that leads to high resistance to existing antibiotics (Davies, 2003). As most virulence traits are non-essential for bacterial survival, inhibition of bacterial virulence is an emerging strategy that does not lead to high-selective pressure and thus is less likely to cause bacterial resistance (Rasko and Sperandio, 2010; Whiteley et al., 2017).

Quorum sensing (QS) is a type of bacterial cell–cell communication through diffusible molecules called autoinducers that regulate collective behaviors such as the secretion of virulence factors, biofilm formation, bioluminescence, and antibiotic resistance (Ng and Bassler, 2009; Rutherford and Bassler, 2012; Papenfort and Bassler, 2016). QS in *P. aeruginosa* consists of three main QS systems: *las*, *rhl*, and *pqs*. Each QS system expresses autoinducer-synthesis genes, such as *lasI*, *rhlI*, and *pqsABCD*, as well as cognate-regulatory genes, including *lasR*, *rhlR*, and *pqsR* (Papenfort and Bassler, 2016). The acyl homoserine lactone (AHL) synthases LasI and RhlI produce N-(3-oxododecanoyl)-l-homoserine lactone (OdDHL) and N-butanoyl homoserine lactone (BHL), respectively, as signaling molecules whereas the quinolone synthase PqsABCD synthesizes 2-heptyl-3-hydroxy-4(1H) quinolone (PQS). The three QS systems are hierarchically regulated. Upon activation by OdDHL, the LasR-OdDHL complex activates the expression of the *rhl* and *pqs* QS systems and directs the gene expression of biofilm components and virulence factors, such as elastase (Lee and Zhang, 2015; Papenfort and Bassler, 2016). The RhlR-BHL complex, in turn, controls many QS-dependent virulence factors, such as rhamnolipid and pyocyanin. The PqsR-PQS complex direct the expression of gene cascades associated with the PQS system and virulence factors, such as pyocyanin and rhamnolipids (Jimenez et al., 2012; Lee and Zhang, 2015). Recently, 2-(2-hydroxyphenyl) thiazole-4-carbaldehyde (IQS), has been reported to be a fourth QS signaling molecule (Lee et al., 2013). The relationship between QS and biofilm formation is not fully understood (Papenfort and Bassler, 2016). The Las system is known to regulate the synthesis of exopolysaccharides, which forms the biofilm matrix (Sakuragi and Kolter, 2007). Rhamnolipids, RhlR-activated exoproducts, have been reported to be involved in the architectural development and dispersal of biofilms (Davey et al., 2003; Boles et al., 2005). Thus, the Las system appears to participate in the biofilm formation, while the Rhl system participates in the biofilm maintenance and dispersal.

LasR is at the top of the QS hierarchy in *P. aeruginosa* and activates the RhlR and PqsR pathways (Latifi et al., 1996; Pesci et al., 1997). Thus, LasR has been a primary target in the development of QS antagonists designed to prevent *P. aeruginosa* infections (Galloway et al., 2011; Welsh and Blackwell, 2016). However, mutations in *lasR* are common in *P. aeruginosa* in the lungs of chronically infected patients (Smith et al., 2006; Hoffman et al., 2009; Bjarnsholt et al. 2010). Notably, in the LasR mutants from chronic patients, RhlR independently expresses QS-regulated phenotypes, such as C4-HSL signaling molecules and virulent factors (Bjarnsholt et al. 2010; Feltner et al., 2016). In contrast, *rhlR* mutations are rare in CF lung infections. It has been reported that RhlR mutants are not viable because RhlR mutants are sensitive to cyanide produced by both LasR and PqsR mutants which are resistant to cyanide (Chen et al., 2019). Thus, for effective treatment of chronic CF infections, RhlR antagonists is needed to prevent the LasR-independent Rhl system, which is a functioning QS system in chronic *P. aeruginosa* infections (Whiteley et al., 2017). However, only a few RhlR antagonists have been reported. Recently, N-cyclopentylbutyramide (CPBA) and 1-(3,4-difluorophenyl)hex-1-yn-3-one (DFPH), which are synthetic derivatives of AHL and 4-gingerol, respectively, were reported to be RhlR antagonists (Eibergen et al., 2015; Boursier et al., 2018; Nam et al., 2020). We have also reported RhlR-selective inhibiting activity of hexyl gallate (HG) which is a synthetic alkyl ester derivative of gallate (Kim et al. 2019).

**Figure 1:**
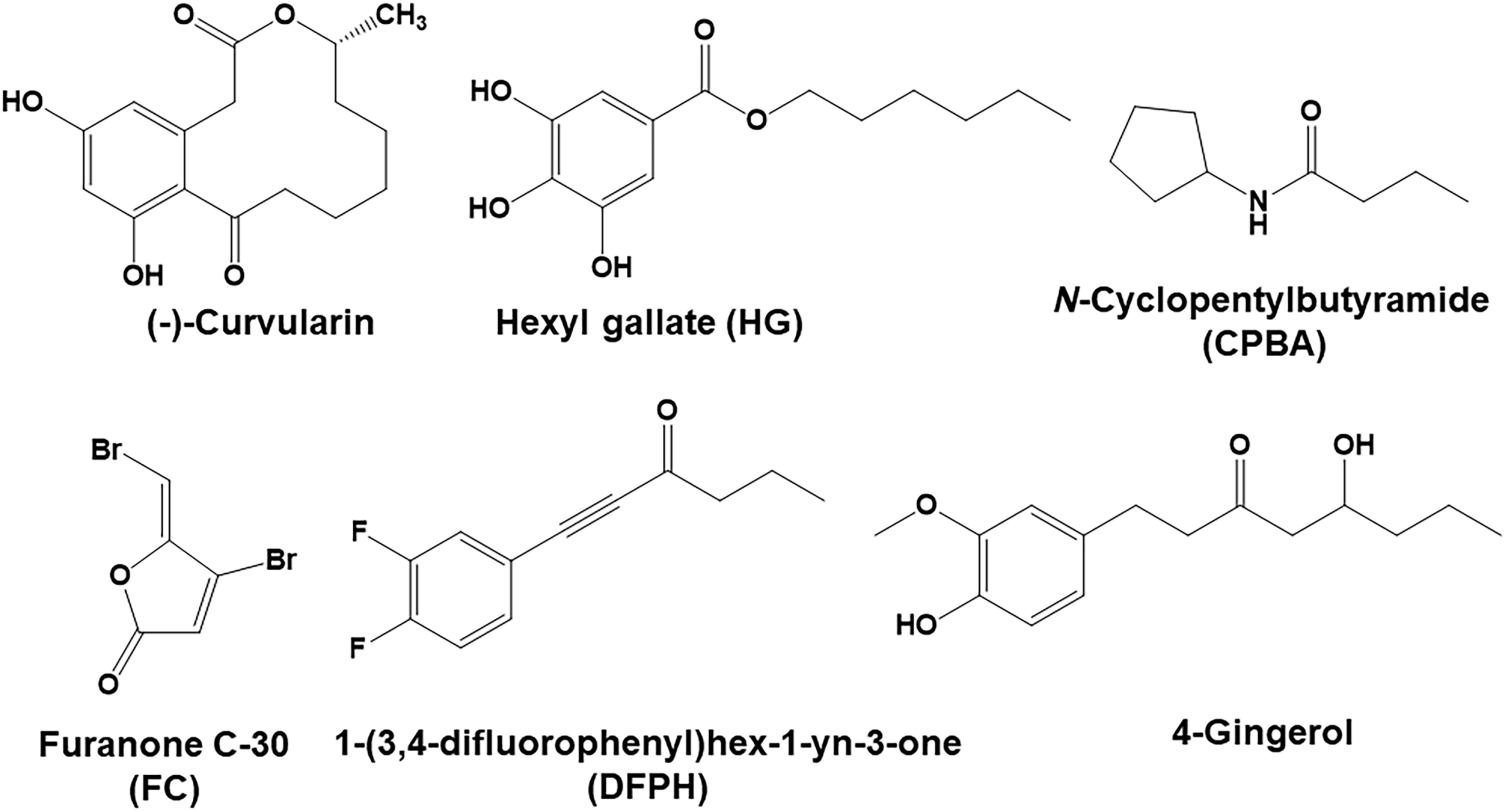
(−)-Curvularin and related compounds.

In this study, while screening for an inhibitor of *P. aeruginosa* pyocyanin production from microbial fermentation extract library, we found that fermentation extracts of a soil fungus, *Phoma macrostoma* FN413, reduced pyocyanin and rhamnolipid production but, interestingly, did not inhibit biofilm formation or elastase production in *P. aeruginosa*. This phenotype was similar to that of HG, but different from that of CPBA and DFPH that also inhibits biofilm formation. This discrepancy led to the isolation of an active compound from culture extracts, and an investigation into the QS antagonizing activity of this compound and the relationship between certain QS and virulent factors. Here, we report that curvularin is a highly RhlR-selective antagonist against *P. aeruginosa* infections, exhibiting a greater effects than that reported for other RhlR antagonists (Figure 1). To the best of our knowledge, curvularin is the first RhlR antagonist to be isolated from natural resources.

## Materials and Methods

### Bacterial Strains and Reagents

The bacterial strains used in this study were *Agrobacterium tumefaciens* NT1, *Chromobacterium violaceum* CV026, *P. aeruginosa* PA14, and *P. aeruginosa* PA14 mutants (Δ*lasR*, Δ*rhlR*, and Δ*pqsR*). All bacterial strains were grown in Luria-Bertani (LB) medium. Furanone C-30 (FC), CPBA, and clofoctol (CF) were purchased from Sigma-Aldrich (St. Louis, MO, United States).

### Molecular Identification of the Fungal Strain FN70413

The genomic DNA from cultured fungal strain FN70413 was extracted using the PureHelix™ Genomic Prep Kit (Catalog #GCTN200, NanoHelix, Daejeon, South Korea). 18S rDNA was amplified from the extracted gDNA by PCR using universal primer pairs of NS1 (5′-GTAGTCATATGCTTGTCTC-3′) and NS8 (5′-TCCGCAGGTTCACCTACGGA-3′). PCR reaction was carried out in 25 μl reaction volume containing 10 ng of gDNA, 2.5 mM MgCl_2,_ 0.5 μM of each primer (Macrogen, Seoul, South Korea), 200 μM of each dNTP, 0.5 U of Ex Taq DNA polymerase, and 1× Ex Taq PCR buffer (Takara, Otsu, Japan). PCR amplification were performed using a thermocycler (Dice Model TP600; Takara) under the following conditions: 94°C for 2 min, followed by 30 cycles of 94°C for 30 s, 45°C for 45 s, and 72°C for 1 min 30s with a final extension of 72°C for 5 min. The amplified PCR product was purified by PCR purification kit (Biofact, Daejeon, South Korea) and sequenced by Macrogen Inc., South Korea. Sequences were annotated and analyzed at BLAT to search the closet homolog.

### Pyocyanin Assay

The production of pyocyanin in *P. aeruginosa* PA14 was measured following a previously reported method with modification (Essar et al., 1990). *P. aeruginosa* PA14 was cultivated in LB medium overnight at 220 rpm at 37°C. The overnight cultures were diluted 100–fold in LB medium, and 0.15-ml aliquots of the diluted culture were dispensed in wells of a 96-well microtiter plate, and treated with test compounds dissolved in dimethyl sulfoxide (DMSO). After incubation at 37°C for 24 h, cell viability was assayed by measuring the optical density (OD) at 600 nm, followed by transfer of the culture medium to 2-mL Eppendorf tubes and centrifugation at 12,000 rpm at 4°C for 10 min. The supernatant was extracted with chloroform, and the chloroform layer was extracted with 0.2 N HCl. The OD of resultant aqueous fraction was measured at 520 nm.

### Rhamnolipid Assay

The production of rhamnolipid in *P. aeruginosa* PA14 was tested as previously described (Boles et al., 2005). After incubation overnight, *P. aeruginosa* PA14 cultures were diluted 100–fold in LB medium, and 5 ml of the diluted culture was dispensed into 50-ml conical tubes and treated with test compounds dissolved in DMSO. After incubation with rotation at 220 rpm at 37°C for 24 h, cell viability was assayed by measuring the OD at 600 nm, and the cultures were then centrifuged at 12,000 rpm at 4°C for 10 min. Then, 500 μl of supernatant was mixed with 500 μl of diethyl ether. The ether fraction was evaporated to dryness and dissolved in 500 μl of deionized water. Then, 100 μl of the water extract was mixed with 900 μl of Orcinol solution [0.19% Orcinol (Sigma) in 53% H_2_SO_4_]. The mixture was boiled for 30 min and cooled at room temperature for 15 min, and then the OD was measured at 421 nm with a microplate reader.

### Elastase Assay

The production of elastase in *P. aeruginosa* PA14 was assayed with Elastin-Congo red as previously described (Rust et al., 1994). *Pseudomonas aeruginosa* PA14 was cultured overnight in LB medium at 37°C with shaking. Then, the cultures were then diluted 1:100 with fresh LB medium, and the diluted culture was added at 0.1 ml/well in a 96-well microtiter plate, followed by treatment with test compounds dissolved in DMSO and incubation for 24 h. After determination of cell viability by measuring the OD at 600 nm and subsequent centrifugation of the culture samples at 4,000 rpm for 10 min, the elastase activity in the culture supernatants was measured.

### Biofilm Assay

Biofilm formation in *P. aeruginosa* PA14 was measured as previously described (O'Toole, 2011). *Pseudomonas aeruginosa* PA14 was cultured overnight and then diluted 100-fold in M63 medium, and the diluted culture was dispensed at 0.1 ml/well in a 96-well polystyrene microplate. Test compounds or DMSO, as the negative control, were added to wells. After incubation at 37°C for 9 h without agitation, unattached cells and medium were removed, and the cells forming the biofilm, which remained attached to the well surface, were stained for 10 min with 120 μl of 0.1% crystal violet. The bound crystal violet was solubilized with 150 μl of 30% acetic acid in water for 15 min. The OD of the eluted crystal violet was measured at 550 nm with a microplate reader.

### QS Competition Assay

An AHL-based QS competition assay was performed using two reporter strains, namely, *A. tumefaciens* NT1 and *C. violaceum* CV026 (Park et al., 2006). Each strain was cultivated in LB medium overnight with a rotation of 220 rpm at 30°C and then diluted 20-fold. One milliliter of the diluted culture was then dispensed into a 15-mL conical tube, and 5 μl of X-gal (20 g/L) and 10 μl of 100 μM OdDHL (Sigma) were added to *A. tumefaciens* NT1 and 10 μl of 50 mM BHL (Cayman, Ann Arbor, MI, United States) was added to *C. violaceum* CV026. Ten microliters of each test compound dissolved in DMSO was added to the culture tubes and then incubated with 180 rpm rotation at 30°C for 36 h. After the cell viability was assayed by measuring the OD at 600 nm with a microtiter ELISA reader, color changes in *C. violaceum* CV026 and *A. tumefaciens* NT1 were measured at 590 and 545 nm, respectively.

### Measurement of QS Signaling Molecules and QS Gene Expression

QS signaling molecule production (Luo et al., 2017) and QS gene expression (Gi et al., 2014) were determined as previously described. *P. aeruginosa* PA14 was cultured and treated with test compounds following the same procedure used for the rhamnolipid assay. To measure three QS signaling molecules, 3 ml of culture supernatant was extracted with 3 ml of ethyl acetate acidified with 0.1% acetic acid, and then, the organic layer was evaporated and the remaining molecules were measured by liquid chromatography with tandem mass spectrometry (LC–MS/MS) with the spectrometer in MRM mode (Supporting Information). To determine QS gene expression, total RNA was extracted with TRIzol reagent (Invitrogen), and cDNA was then synthesized for real-time quantitative PCR (RT–qPCR) detection of target gene expression, followed by measurement *via* RT–qPCR on a Bio–Rad CFX-96 real-time PCR system (Bio–Rad, Hercules, CA, United States) with the primers listed in Supplementary Table 1. mRNA expression was normalized to the expression of the endogenous *rpoD* gene.

### *Caenorhabditis elegans* Life Span Assay

A *P. aeruginosa*-*C. elegans* infection assay was performed as previously described (O'Loughlin et al., 2013). *Caenorhabditis elegans* nematodes were propagated at 20°C for 48 h on nematode growth medium (NGM) plates containing a lawn of *E. coli* OP50 as a food source. Killing and control plates were established by spreading the overnight cultures of PA14 and OP50 cells, respectively, on a 35-mm petri plate with 4 ml of PGS agar. Test compounds dissolved in DMSO or DMSO as a negative control were added to the killing plates. The plates were then incubated at 37°C for 24 h to make a bacterial lawn and then transferred for incubation at 23°C for 24 h. Thirty L4-stage worms were placed on each plate and incubated at room temperature. The surviving nematodes were counted every 5 h for 30 h.

## Results

### Screening and Isolation of an Inhibitor of Pyocyanin Production That Displayed a Phenotype Similar to a RhlR Antagonist

Screening of 3,696 microbial fermentation extracts were performed for inhibitors of pyocyanin production in *P. aeruginosa* PA14 that resulted in selection of 25 strains. Among these identified strains, the fermentation extracts of only the fungal strain FN70413, which was isolated from forest soil, inhibited the production of pyocyanin and rhamnolipid without affecting cell viability; interestingly, it did not inhibit biofilm formation by PA14. This phenotype was similar to that of HG, which we have previously reported to an RhlR-selective antagonist, but differs from that of the other reported RhlR antagonists, namely, CPBA and DFPH, which have been reported to exhibit antibiofilm activity. Additionally, the extracts inhibited pyocyanin production by a *lasR* mutant of the PA14 strain, and this inhibition was reversed when cultures were supplemented with exogenous BHL, but not with OdDHL or PQS (data not shown), suggesting that RhlR was selectively inhibited by factors in the extract fraction (Kim et al. 2019). Thus, an active compound was isolated from the culture extracts of the fungal strain *via* bioassay-guided fractionation and then identified as (−)-curvularin by mass and NMR spectral analysis; in addition, a specific rotation value ([α]_D_, −23.8, *c* 0.1, MeOH) was obtained, and it was almost the same as the value ([α]_D_, −23.4, *c* 0.1, MeOH) reported in the literature (Allu et al., 2019; Supplementary Figures 1, 2; Supplementary Table 2). The purity of isolated curvularin was determined to be 98.3% at 220 nm by analytical HPLC (Supplementary Figure 3). The fungal strain FN70413 was identified as *Phoma macrostoma* using phylogenetic analysis based on 18S rDNA.

### Effect of Curvularin on Virulence Factor Production and Biofilm Formation

**Figure 2:**
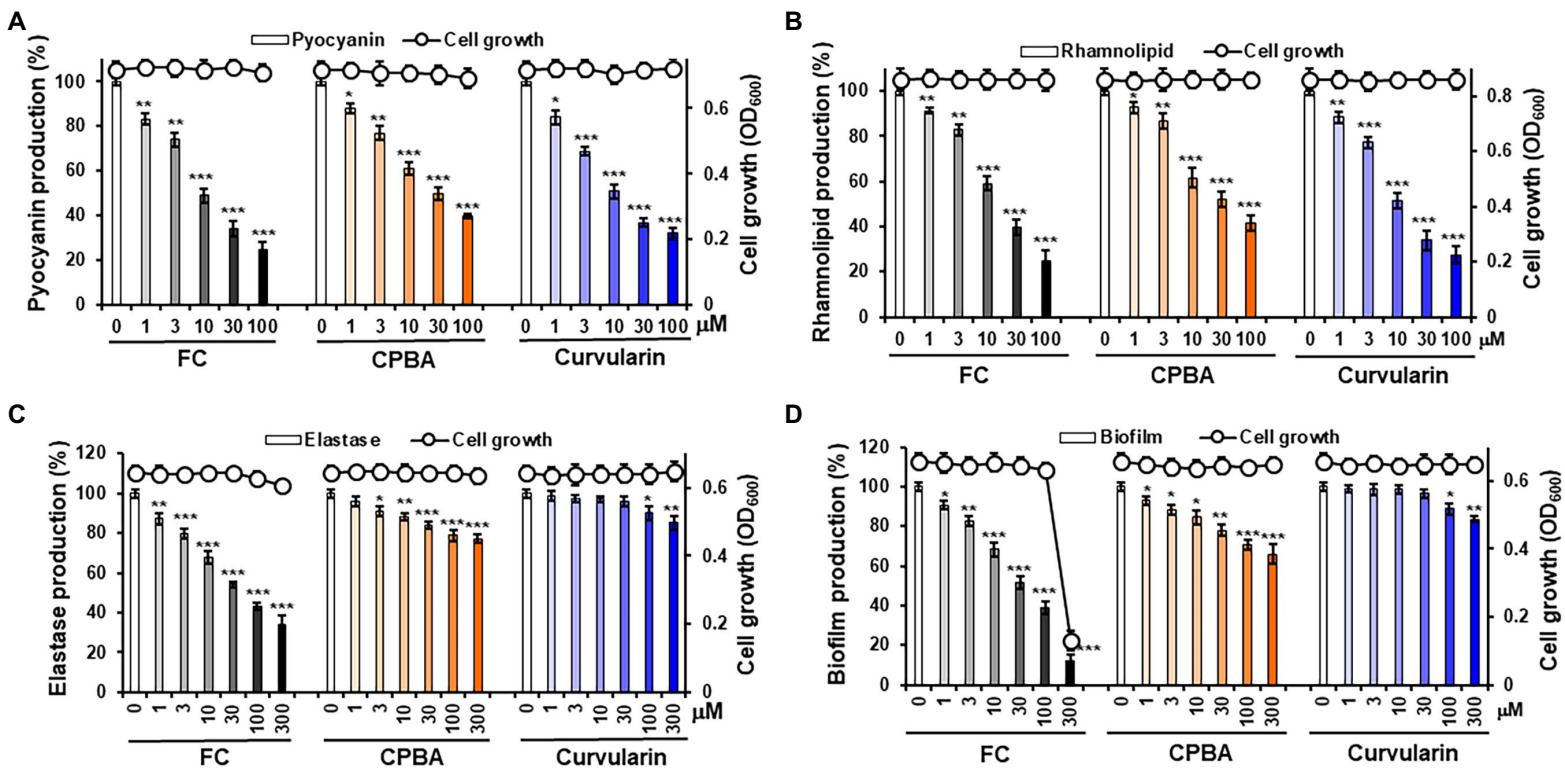
Effects of curvularin on *P. aeruginosa* virulence factor production and biofilm formation. **(A–C)** Effects of curvularin on virulence factor production and cell viability in *P. aeruginosa* PA14. After PA14 cells were grown in LB medium containing various concentrations of curvularin for 24 h, cell density was measured at 600 nm and pyocyanin and rhamnolipid and elastase activity in the culture supernatants were then determined. **(D)** Effects of curvularin on *P. aeruginosa* biofilm formation and cell viability. PA14 biofilms were grown in the presence of curvularin for 9 h, followed by the measurement of planktonic cell density at 600 nm. The biofilm cells attached to the well surface were assayed using crystal violet staining. Furanone C-30 (FC) and N-cyclopentylbutyramide (CPBA) are known antagonists of LasR and RhlR, respectively. Three independent experiments were performed in triplicate, and the mean ± SD values are presented in each bar. *p < 0.01; **p < 0.001; and ^***^p < 0.0001 versus untreated cells.

To determine whether curvularin selectively inhibits virulence factor production, the effects of curvularin on the production of virulence factors, including pyocyanin, rhamnolipid and elastase, in PA14 cells were investigated. The effect of curvularin on cell viability was also examined using an optical-density-based assay of each virulence factor. Curvularin inhibited the production of pyocyanin in a concentration-dependent manner without affecting the cell growth of the PA14 strain (Figure 2A). Treatment with 3 and 30 μM curvularin for 24 h significantly inhibited pyocyanin production, by 31.3% and 63.2%, respectively, and this inhibitory effect was approximately three times greater than that induced by 10 and 100 μM CPBA, which reduced production by 39.2% and 60.2%, respectively, compared with pyocyanin production in untreated PA14 cells. Similarly, rhamnolipid production was significantly inhibited, by 22.8% and 66.1%, respectively, in the presence of 3 and 30 μM curvularin for 24 h, and this effect was approximately threefold greater than that induced by 10 and 100 μM CPBA, which inhibited rhamnolipid production by 38.5% and 58.4%, respectively, compared with the rhamnolipid production by the untreated cells (Figure 2B). Similarly, curvularin also showed more than threefold greater inhibition of pyocyanin and rhamnolipid production than the inhibition induced by HG (Supplementary Figures 4A,B). FC, a known LasR antagonist, exhibited inhibitory activity similar to that of curvularin.

Interestingly, at low concentrations of 1–30 μM curvularin did not inhibit elastase production, although pyocyanin and rhamnolipid production was dramatically reduced, whereas low–concentration FC, as a positive control, showed a strong inhibition as expected. However, treatment with curvularin at high concentrations, more than 100 μM, led to very weak inhibition of elastase production (Figure 2C). Elastase production by PA14 cells in the presence of 100 and 300 μM curvularin for 24 h was inhibited by 8.1% and 11.3%, respectively, compared to the production by untreated PA14 cells. Interestingly, at low concentrations, CPBA significantly inhibited elastase production. For example, elastase production in the presence of 3 and 30 μM CPBA for 24 h was inhibited by 8.9% and 16.1%, respectively, compared to the production by untreated PA14 cells. Similar to its effects on elastase, low-level curvularin did not affect biofilm formation, and at a high concentration, curvularin showed very weak biofilm-inhibitory ability. In contrast, low-concentration CPBA inhibited biofilm production. For example, 100 μM curvularin inhibited biofilm formation by approximately 10%, whereas only 3 μM CPBA inhibited biofilm formation by 10%. HG showed antibacterial activity in the elastase assay but did not affect biofilm formation at all as reported previously (Kim et al., 2019; Supplementary Figures 4C,D). As a positive control, low-concentration FC profoundly inhibited biofilm formation. Given previous reports indicating that pyocyanin and rhamnolipid are RhlR-directed exoproducts and that biofilm formation is regulated by the Las system, these results suggest that curvularin at low concentrations might selectively antagonize RhlR.

### Inhibitory Effects of Curvularin on QS

To test whether curvularin selectively blocks QS receptors, an AHL-based *in vitro* QS competition assay was performed using the two reporter strains *A. tumefaciens* NT1 and *C. violaceum* CV026 (Park et al., 2006), as reported previously (Kim et al., 2018). Briefly, *A. tumefaciens* NT1 contains the TraR receptor gene fused to the *lacZ* reporter gene. The TraR receptor can sense long-chain AHLs, such as OdDHL, leading to the synthesis of a cyan pigment (Zhang et al., 1993). The *C. violaceum* CV026 strain carries the CviR receptor, which can sense short-chain AHLs, such as BHL, resulting in the production of a purple pigment violacein (McClean et al., 1997). Because the TraR and CviR receptors are homologs of the LasR and RhlR receptors, respectively, a competitive binding assay for the LasR and RhlR receptors was performed using these reporter strains (Kim et al., 2015; Srivastava et al., 2015).

**Figure 3:**
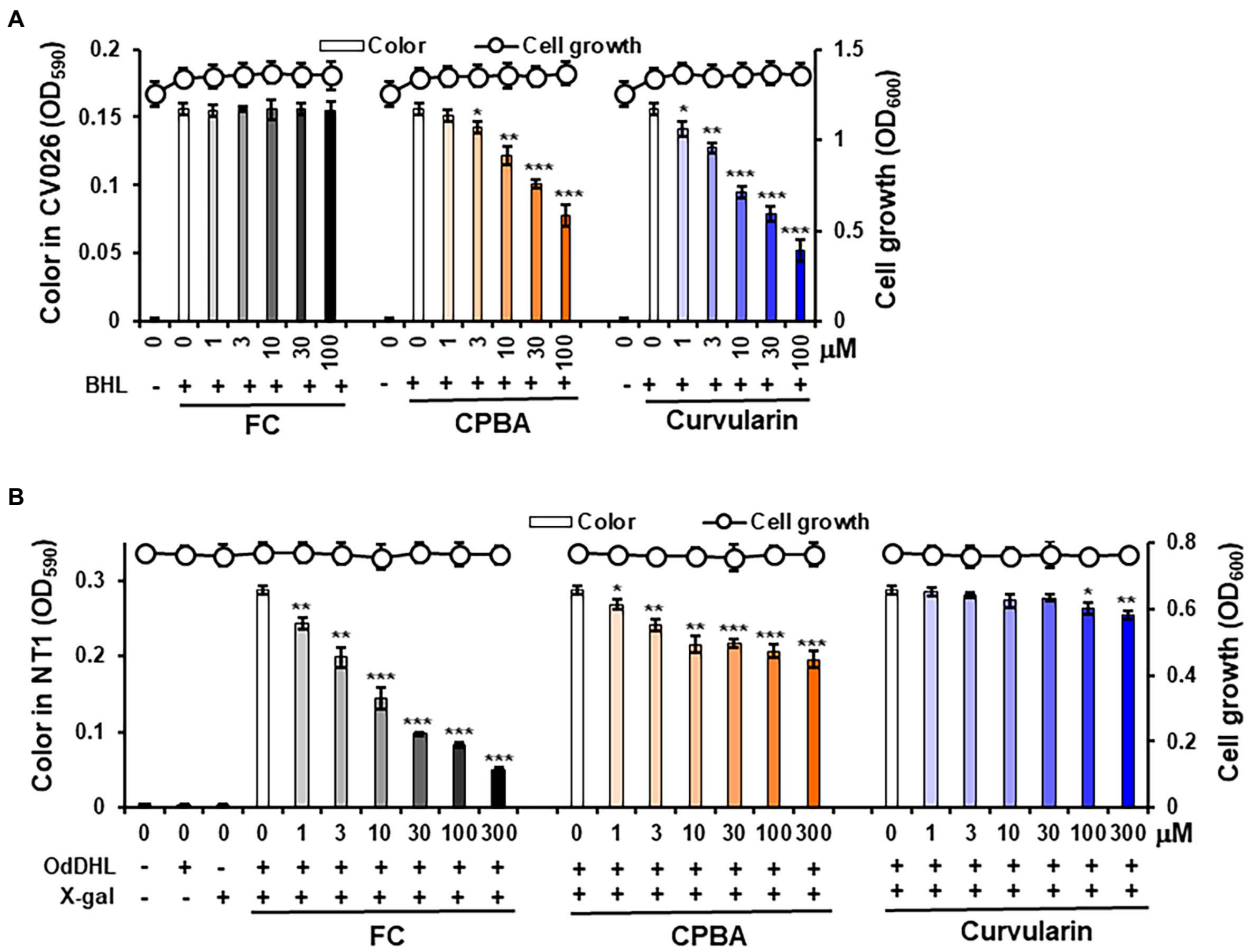
Effects of curvularin on QS. QS competition against N-butanoyl homoserine lactone (BHL) and N-(3-oxododecanoyl)-l-homoserine lactone (OdDHL) was performed using the reporter strains *Agrobacterium tumefaciens* NT1 and *C. violaceum* CV026, respectively. After 1 μM OdDHL and 500 μM BHL were added to the NT1 and CV026 cultures, respectively, with various concentrations of curvularin and incubated for 24 h, cell growth at 600 nm and color change intensity were measured. **(A)** Curvularin—BHL competition. **(B)** Curvularin—OdDHL competition. Furanone C-30 (FC) and N-cyclopentylbutyramide (CPBA) are known antagonists of LasR and RhlR, respectively. The data are expressed as the mean ± SD values of three independent experiments performed in triplicate. *, *p* < 0.01; **, *p* < 0.001; and ^***^, *p* < 0.0001 versus untreated cells.

Violacein pigment was produced in the CV026 cultures only when BHL was added, as expected (Figure 3A). Treatment with 1–100 μM curvularin inhibited the production of pigment without affecting cell growth. Treatment with 3 and 30 μM curvularin inhibited pigment production by 18.6% and 50.0%, respectively, approximately threefold greater than the effect of CPBA, with 22.4% and 50.7% inhibition at 10 and 100 μM treatment, respectively. Similarly, curvularin induced more than a threefold greater inhibition of violacein production than that induced by HG (Supplementary Figure 5A). FC, a LasR antagonist, did not inhibit pigment production, as expected. In contrast, in an NT1 culture, low-concentration curvularin did not inhibit pigment production, and at a concentration greater than100 μM, it showed very weak pigment production inhibition (Figure 3B); curvularin showed inhibition of 2.5% and 11.5% only at high concentration levels, that is at 100 and 300 μM, respectively, whereas as the positive control, FC at concentrations as low as 1 μM showed a strong inhibitory effect, as expected. However, 3 and 30 μM CPBA inhibited pigment production in NT1 cells by 16.0% and 24.5%, respectively. The effects of HG on LasR could not be determined using NT1 cells due to its antibacterial activity as reported previously (Kim et al. 2019; Supplementary Figure 5B). These results showed that low concentrations (1–30 μM) of curvularin antagonized only the binding of BHL to its cognate receptor RhlR, which showed threefold higher potency than that exhibited by CPBA or HG, and at concentrations greater than100 μM, curvularin weakly inhibited the binding of OdDHL to LasR, whereas CPBA at low concentrations inhibited OdDHL binding to LasR. These results indicate that low-concentration curvularin is an RhlR antagonist.

### Effect of Curvularin on QS Signaling Molecules Production and QS Gene Expression

**Figure 4:**
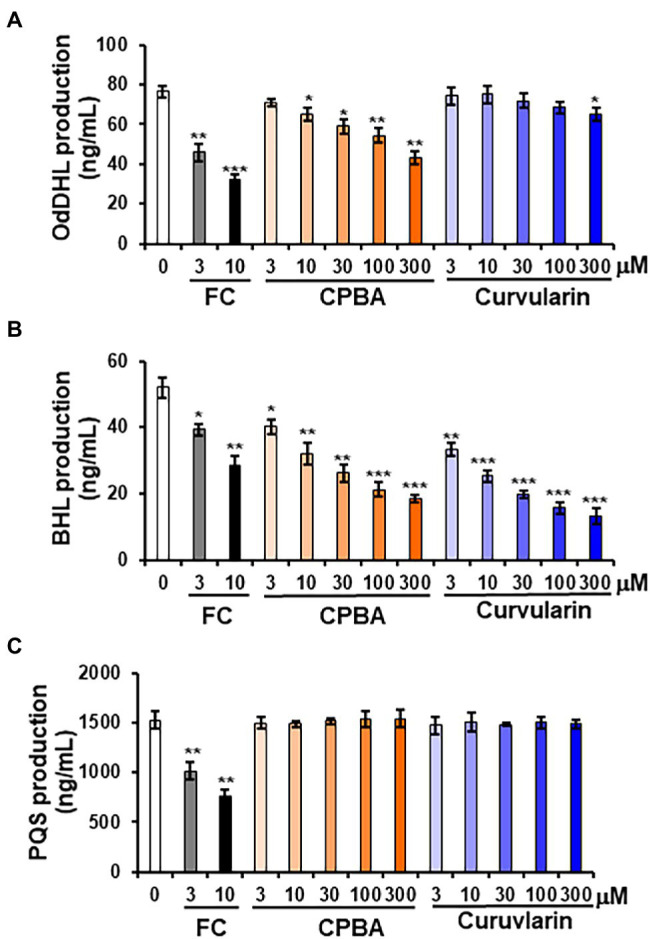
Effects of curvularin on QS signaling molecule production. PA14 cells were cultured in LB medium in the presence of curvularin for 12 h. The three main QS molecules, namely, OdDHL **(A)**, BHL **(B)**, and PQS **(C)**, were extracted from the culture supernatants and quantitatively analyzed by LC–MS/MS. FC and CPBA are known antagonists of LasR and RhlR, respectively. Three independent experiments were performed in triplicate, and the mean ± SD values are presented in each bar. *p < 0.01; **p < 0.001; and ^***^p < 0.0001 versus DMSO treatment.

To determine whether curvularin selectively affects QS signaling, the effects of curvularin on the production of the AHL and PQS molecules in PA14 were investigated. QS signaling molecules such as OdDHL, BHL, and PQS were quantitatively analyzed by LC–MS/MS (Figure 4). In the supernatants of untreated PA14 cultured for 12-h, 76.37 ± 2.65 ng/ml, 51.93 ± 0.95 ng/ml, and 1500.26 ± 46.52 ng/ml of OdDHL, BHL, and PQS, respectively, were produced. Consistent with the RhlR-specific antagonizing activity of low-concentration curvularin in the QS competition assays, only BHL production was reduced by 3–30 μM curvularin in a dose-dependent manner (Figure 4B), whereas OdDHL production was reduced by 15% only by high-concentration (more than 300 μM) curvularin, and PQS production was not affected (Figures 4A,C). As the control, low-concentration CPBA inhibited not only BHL production but also weakly inhibited OdDHL production, which was consistent with the results of the QS competition assays. Additionally, in agreement with its higher RhlR-antagonizing activity, curvularin exhibited threefold greater inhibition of BHL production than CPBA or HG (Supplementary Figure 6B).

**Figure 5:**
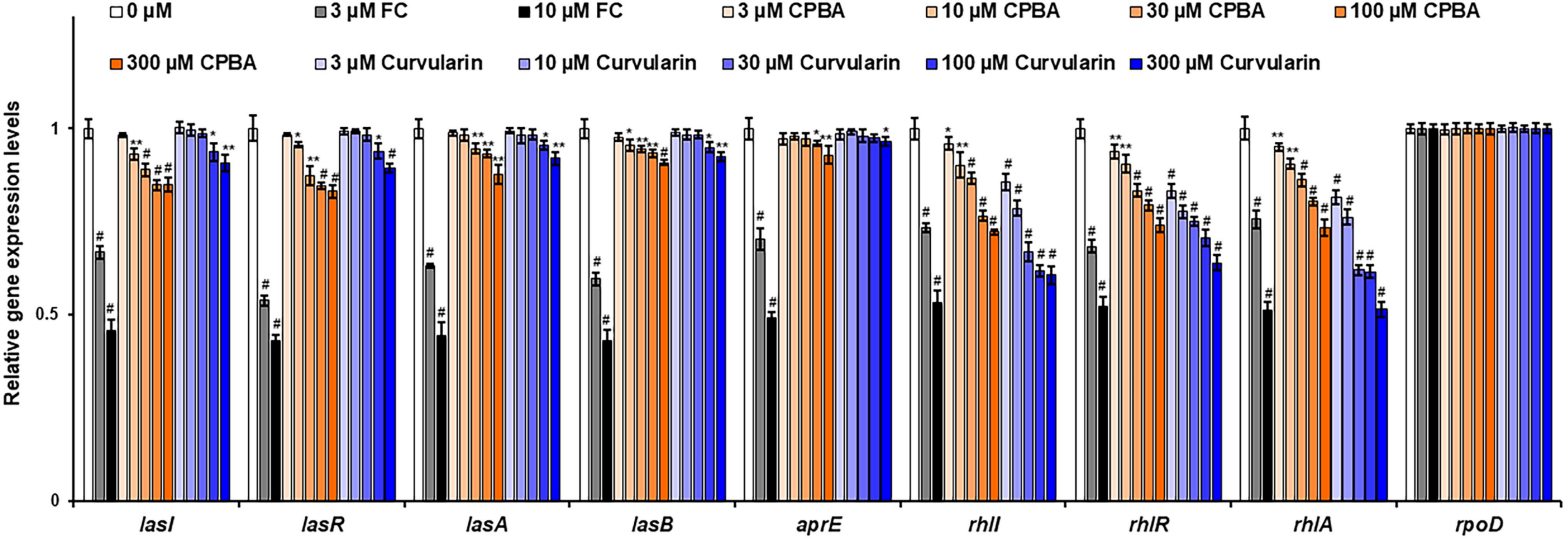
Effects of curvularin on QS gene expression. PA14 cells were cultured in LB medium containing different concentrations of curvularin for 12 h. The effects of curvularin on QS gene expression were measured by RT-qPCR. FC and CPBA are known antagonists of LasR and RhlR, respectively. The experiment shown is representative of three independent experiments performed in triplicate and the mean ± SD values are presented in each bar. *p < 0.05; **p < 0.01; and ^#^*p* < 0.001 versus DMSO treatment.

The effects of curvularin on QS gene expression were also investigated by RT–qPCR. The expression levels of QS regulatory genes (*lasI*, *lasR*, *rhlI*, *rhlR*, *pqsA*, *phnB*, *pqsH*, and *pqsR*) and key QS-controlled genes (*lasA, lasB, aprE*, *rhlA*, and *phzA2*) in PA14 cells were measured (Figure 5; Supplementary Figure 7). Low-concentration curvularin selectively inhibited *rhl*-related genes, as expected. Transcription of the *rhlI*, *rhlR*, and *rhlA* genes was selectively inhibited by 3–30 μM curvularin, and transcription of *las*-related genes (*lasI*, *lasR*, *lasA*, and *lasB*) was weakly suppressed only by curvularin at a concentration higher than 100 μM. Similar to the results of the QS receptor and signaling molecule production assays, CPBA showed little selective inhibitory effect on the transcription of *rhl*-related genes, and both CPBA and HG inhibited the transcription at a rate threefold less than that of curvularin (Supplementary Figure 8). Transcription of *pqs*-related genes, except for the phenazine synthesis gene *phA2*, was largely unaffected by curvularin at low concentrations. As the control, FC inhibited all QS gene transcription, as expected.

Taken together, these results support the supposition that curvularin at low concentrations shows RhlR-antagonizing activity and that its inhibitory effect is greater and more selective than that of CPBA or HG.

### Low-Concentration Curvularin Inhibits Pyocyanin and Rhamnolipid Production by Antagonizing Only RhlR

**Figure 6:**
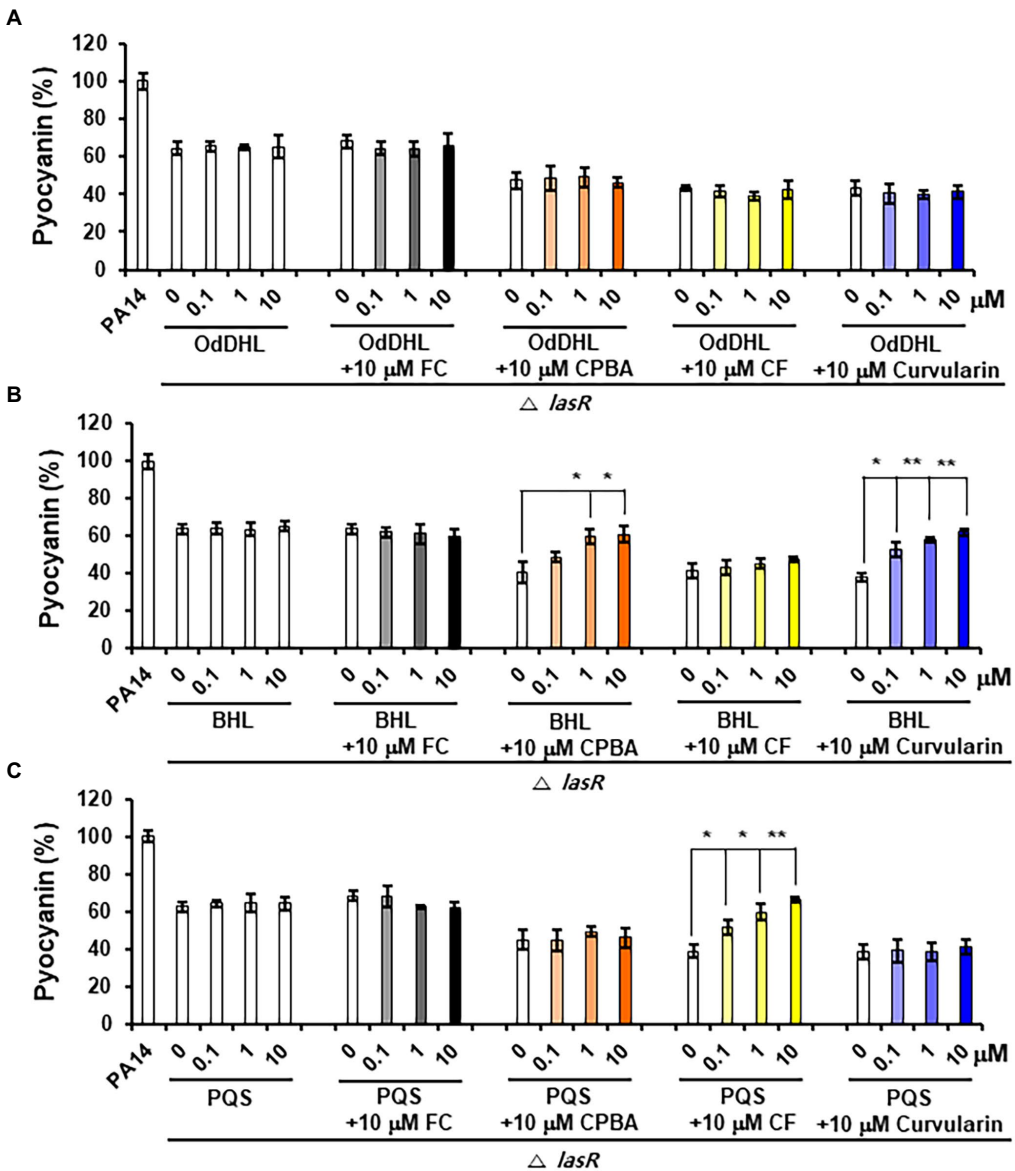
Effects of exogenous QS ligands on curvularin-mediated inhibition of pyocyanin production in the Δ*lasR* mutant. Pyocyanin production in the Δ*rhlR* mutant cultured with curvularin (10 μM) in the presence or absence of different concentrations of OdDHL **(A)**, BHL **(B)**, or PQS **(C)** for 18 h. FC, CPBA, and clofoctol (CF) are known antagonists of LasR, RhlR, and PqsR, respectively. The data are representative of three independent experiments performed in triplicate and expressed as the mean ± SD values in each bar. *p < 0.01 and **p < 0.001 versus DMSO treatment.

**Figure 7:**
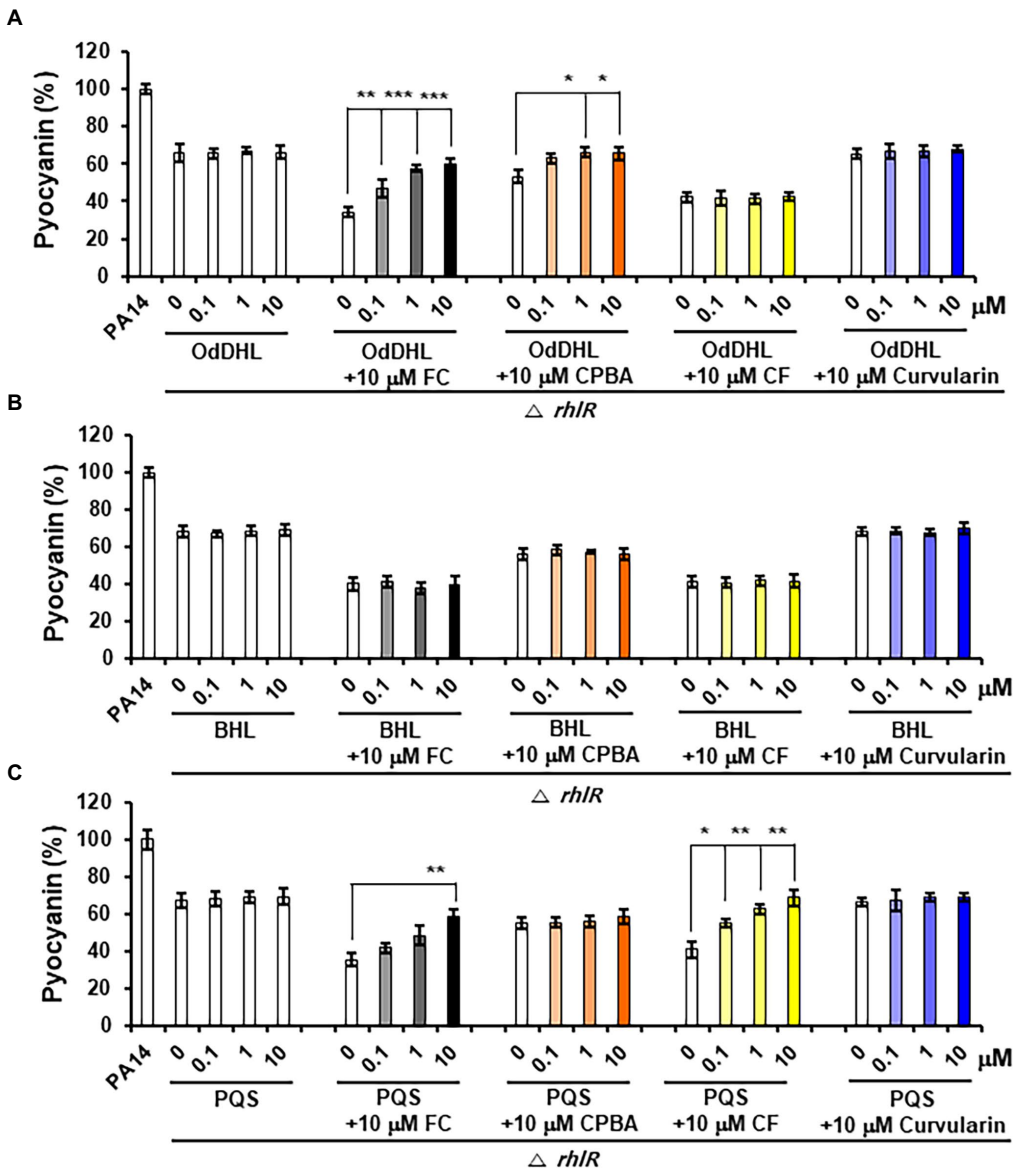
Effects of exogenous QS ligands on curvularin-mediated inhibition of pyocyanin production in the Δ*rhlR* mutant. Pyocyanin production in the Δ*rhlR* mutant cultured with curvularin (10 μM) in the presence or absence of different concentrations of OdDHL **(A)**, BHL **(B)**, or PQS **(C)** for 18 h. FC, CPBA, and CF are known antagonists of LasR, RhlR, and PqsR, respectively. The data are representative of three independent experiments performed in triplicate and expressed as the mean ± SD values in each bar. *p < 0.01; **p < 0.001; and ***p < 0.0001 versus DMSO treatment.

Pyocyanin and rhamnolipid synthesis is activated by both the Rhl and PQS systems (Lee and Zhang, 2015). Moreover, RhlR has been reported to direct the production of virulence factors through the RhlI-independent pathway using an alternative ligand derived from PqsE in the PQS system (Mukherjee et al., 2017). To determine whether the inhibition of pyocyanin and rhamnolipid production by curvularin is mediated exclusively through the antagonization of RhlR and to determine whether this effect is RhlI (BHL) dependent, the effects of exogenous QS ligands on low-concentration curvularin-mediated inhibition of pyocyanin or rhamnolipid production in QS mutants were investigated. In the *lasR* mutant of the PA14 strain, in which pyocyanin production is regulated by the Rhl and PQS systems, curvularin inhibited pyocyanin production, and this effect was reversed by supplementation with exogenous BHL, but not with OdDHL or PQS, demonstrating that low-concentration curvularin exerted its inhibitory effect by preventing BHL-mediated activation of RhlR (Figure 6B). As the control, FC did not inhibit pyocyanin production as expected (Figure 6A), whereas CPBA and CF, which are RhlR and Pqs antagonists, respectively, inhibited pyocyanin production; this effect was revered by the addition of exogenous BHL and PQS, respectively, as expected (Figures 6B,C). In a *rhlR* mutant in which pyocyanin production is controlled by the Las and PQS systems (Figure 7), curvularin lost its activity, demonstrating that curvularin exhibited inhibition by acting on only RhlR. In contrast, FC and CF, positive controls, inhibited pyocyanin production, which was reversed by exogenous supplementation with both OdDHL and PQS and with PQS, respectively, as expected (Figures 7A,C). On the other hand, CPBA weakly inhibited pyocyanin production, and its effect was reversed by the addition of OdDHL, similar to the results observed with FC treatment (Figure 7A), corroborating the finding that low-concentration CPBA acts on both LasR and RhlR. Because the *pqsR* mutant did not produce pyocyanin as previously reported (Cao et al., 2001; Diggle et al., 2003), rhamnolipid production in a *pqsR* mutant, which is regulated by the Las and Rhl systems, was analyzed (Supplementary Figure 9). Curvularin inhibited rhamnolipid production, and this effect was reversed by treatment with exogenous BHL, but not with OdDHL or PQS, similar to its effect on the *lasR* mutant (Supplementary Figure 9B). FC as the positive control inhibited rhamnolipid production, and this effect was reversed by treatment with exogenous OdDHL and BHL, whereas CF, the negative control, did not have a similar effect, as expected. CPBA inhibited rhamnolipid production, which was reversed by the addition of OdDHL and BHL, similar to the effect of FC (Supplementary Figures 9A,B). Overall, these results indicate that low-concentration curvularin inhibited pyocyanin and rhamnolipid production by antagonizing only BHL-activated RhlR, whereas CPBA inhibited this production by antagonizing both RhlR and LasR, although weakly.

### Reduction in Biofilm Formation and Elastase Production Is Due to Inhibition of LasR

**Figure 8:**
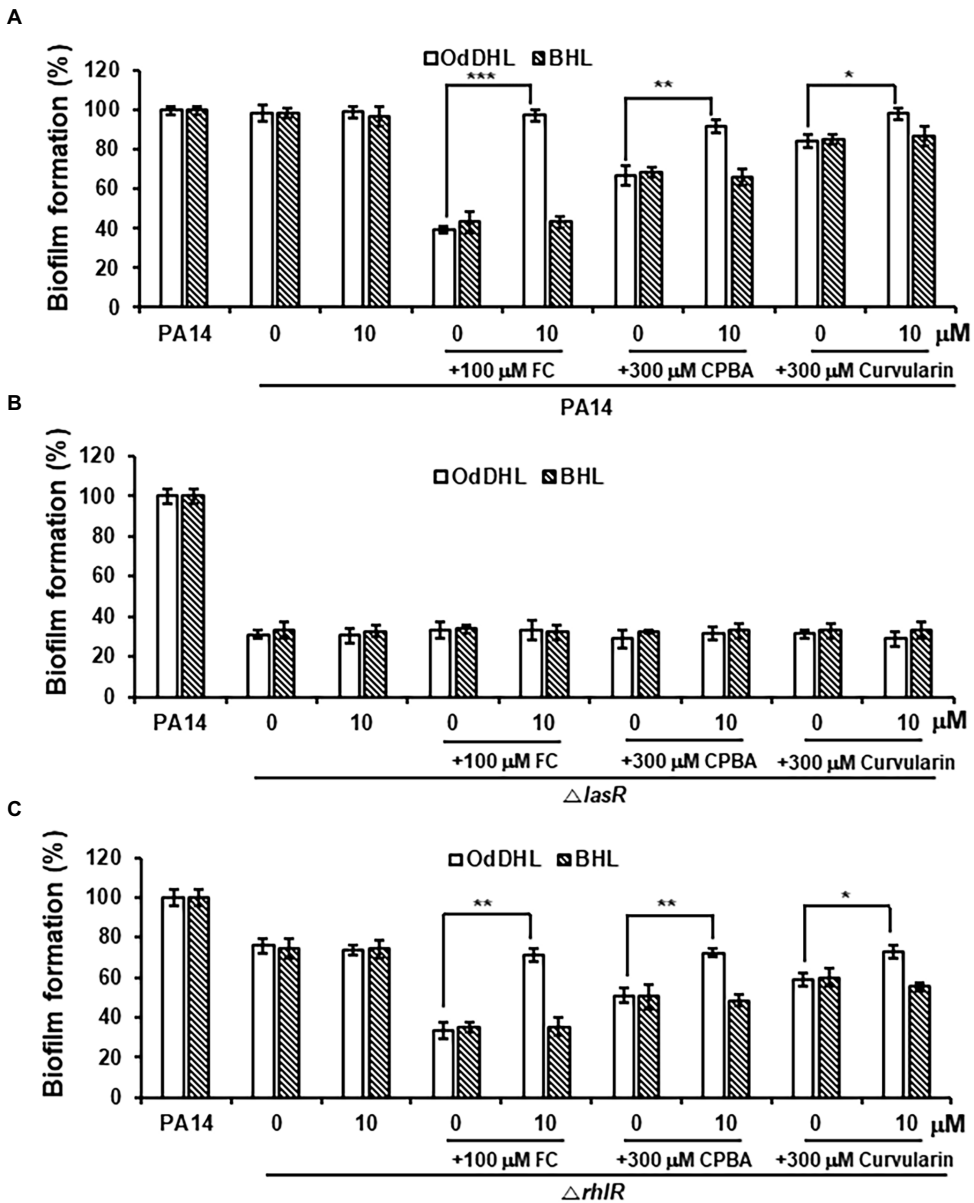
Effects of exogenous QS ligands on curvularin-mediated inhibition of biofilm formation in Δ*lasR* and Δ*rhlR* mutants. Biofilm formation in wild-type PA14 **(A)**, the Δ*lasR* mutant **(B)**, and the Δ*rhlR* mutant **(C)** cultured with curvularin (300 μM) in the presence or absence of different concentrations of OdDHL or BHL for 18 h. FC and CPBA are known antagonists of LasR and RhlR, respectively. The data are representative of three independent experiments performed in triplicate and expressed as the mean ± SD values in each bar. *p < 0.01; **p < 0.001; and ***p < 0.0001 versus DMSO treatment.

After treatment with high-concentration curvularin (more than 100 μM), biofilm formation and elastase production were negligibly inhibited, each by ~10% (Figures 2C,D). Additionally, a very weak inhibition of LasR was observed in the QS competition, QS signaling molecules, and QS gene expression assays with concentrations of curvularin higher than 100 μM. Formation of *P. aeruginosa* biofilms is regulated by the Las system (Davies et al., 1998; Sakuragi and Kolter, 2007), but the regulatory mechanism of elastase synthesis is the Las system and, to a lesser degree, the Rhl system (Ochsner et al., 1994; Pearson et al., 1997). To test whether the weak reduction in biofilm formation and elastase production at high curvularin concentrations is due to the corresponding curvularin inhibition of LasR, the effects of exogenous QS ligands on the curvularin-mediated inhibition of biofilm formation and elastase production were investigated. In wild-type PA14, inhibition of biofilm formation by FC, the positive control, was reversed by supplementation with exogenous OdDHL, but not BHL, as expected (Figure 8A). Similarly, curvularin and CPBA treatment at 300 μM inhibited biofilm formation by 15.6% and 33.2%, respectively, which was reversed by the exogenous addition of OdDHL, but not BHL, suggesting that the reduction in biofilm formation was due to the inhibition of the OdDHL-mediated activation of LasR (Figure 8A). This outcome was confirmed with experiments on QS mutants. In the *lasR* mutant, all tested compounds failed to inhibit biofilm formation, demonstrating that, similar to FC, curvularin and CPBA act on LasR to inhibit biofilm formation (Figure 8B). In a *rhlR* mutant, all tested compounds had the same effect as they did in the wild-type strain, demonstrating that curvularin and CPBA targeted LasR, not RhlR, to inhibit of biofilm formation (Figure 8C). For elastase production, the same results were obtained (Supplementary Figure 10). Taken together, these data indicate that the negligible inhibition of biofilm formation and elastase production induced by the high concentration of curvularin was due to its corresponding inhibition of LasR.

### Confirmation of the RhlR Antagonistic Activity of Low-Concentration Curvularin in *Pseudomonas aeruginosa* PAO1

To confirm the RhlR antagonistic activity of low-concentration curvularin in another *P. aeruginosa*, the effects of exogenous QS ligands on the curvularin-induced inhibition of virulent factor production and biofilm formation were investigated in PAO1 strain. First, the effects of curvularin on virulence factor production and biofilm formation were tested in PAO1. Curvularin potently inhibited production of pyocyanin and rhamnolipid with around threefold higher inhibition than CPBA, whereas biofilm formation was weakly inhibited by only high-concentration curvularin, like in PA14 (Supplementary Figure 11). Interestingly, curvularin inhibited elastase production at both low and high concentrations unlike in PA14 which suggested a differential regulation of elastase between PA14 and PAO1. Exogenous supplementation of BHL (0.1–10 μM), not OdDHL and PQS, reversed the inhibition of pyocyanin and rhamnolipid production by low-concentration curvularin in a dose-dependent manner (Supplementary Figures 12, 13). In contrast, the inhibition of pyocyanin and rhamnolipid production by CPBA was reversed by exogenous addition of OdDHL and BHL. As controls, the inhibition of pyocyanin and rhamnolipid production by FC and CF was reversed by supplementation of the three ligands and PQS, respectively, as expected. In contrast, the inhibition of biofilm formation by high-concentration curvularin was reversed by supplementation of both OdDHL and BHL (Supplementary Figure 14A). These results indicated the RhlR-selective antagonism of low-concentration curvularin in PAO1 like in PA14. Additionally, it was confirmed by analyzing QS signaling molecules production and QS gene expression. Low-concentration curvularin selectively inhibited the production of BHL and the transcription of *rhl*-related genes (Supplementary Figures 15, 16). Taken together, these results corroborated that low-concentration curvularin selectively antagonized RhlR in PAO1 like in PA14.

### Antivirulence Activity of Curvularin in a *Caenorhabditis elegans* Infection Model

**Figure 9:**
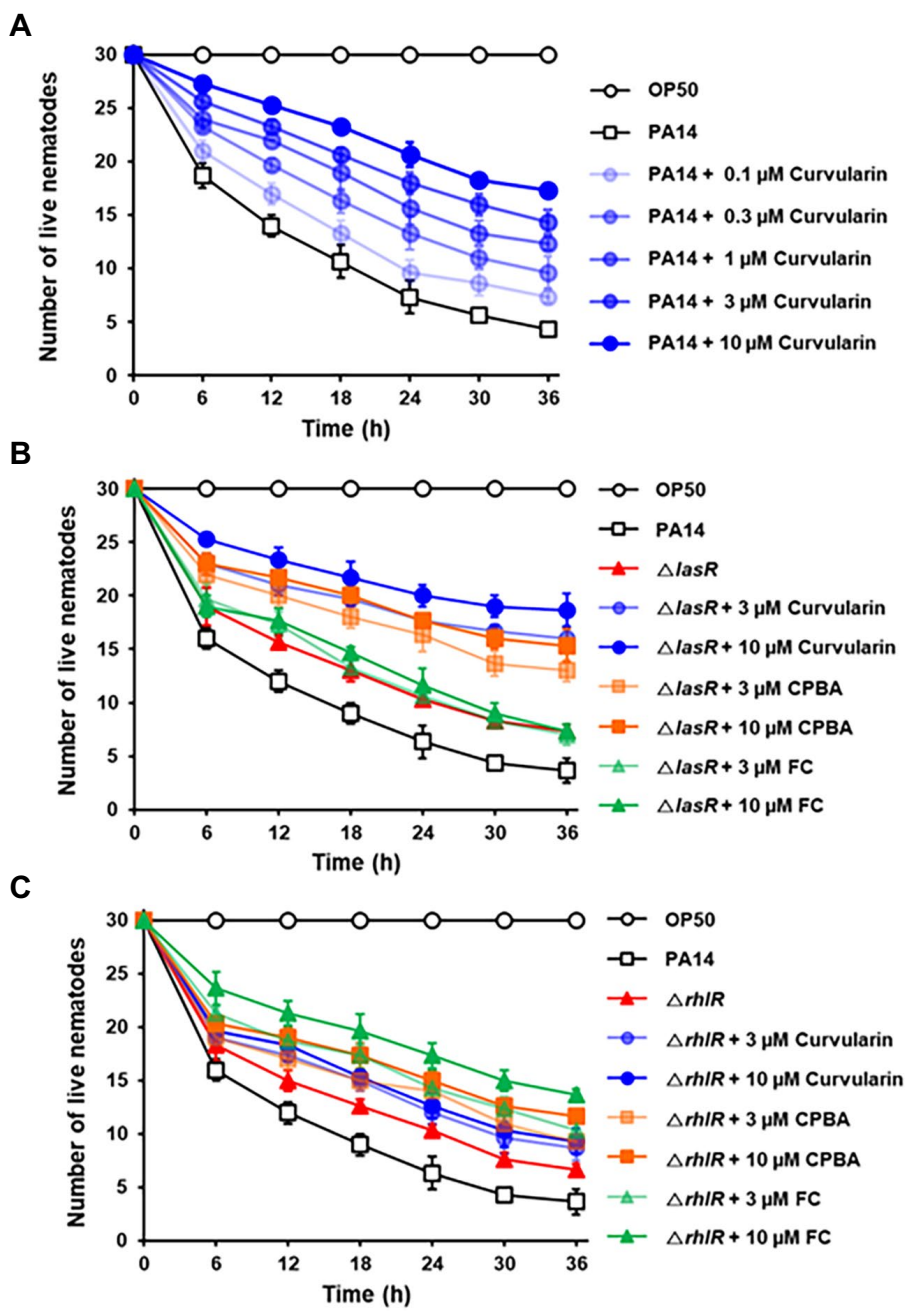
*In vivo* antivirulence activity of curvularin. Antivirulence activity of curvularin in a *C. elegans* infection model. Thirty worms were introduced on lawns of *E. coli* OP50, wild-type PA14 **(A)**, Δ*lasR* mutant **(B)**, or Δ*rhlR* mutant **(C)** on plates in the presence of different concentrations of curvularin. Live nematodes were counted every 5 h for 30 h. FC and CPBA are known antagonists of LasR and RhlR, respectively. Two independent experiments were performed in triplicate, and the mean ± SD values are displayed in each graph.

The effects of curvularin on the *in vivo* virulence of *P. aeruginosa* were examined using a *C. elegans* fast-kill infection assay. *Caenorhabditis elegans* rapidly died when fed with *P. aeruginosa* PA14, as evidenced by the death of 85.6% of the nematodes 36 h postinfection (Figure 9). Treatment with curvularin (0.1–10 μM) and *P. aeruginosa* at the same time prevented the death of the nematodes in a dose-dependent manner. Nematode death significantly decreased by 31.1% and 50.6% after curvularin treatment of 1 and 10 μM, respectively (Figure 9A). Additionally, the protective effects of curvularin was 1.70- and 2.34-fold greater than those of FC and CPBA, respectively, at 36 h treatment (Supplementary Figure 17). These results clearly indicated that curvularin protected *C. elegans* against infection with *P. aeruginosa*. We then tested whether curvularin prevents the *in vivo* virulence of the *lasR* mutant toward *C. elegans*. When infected with the *lasR* mutant, 75.6% of the nematodes died after 36 h. As the control, the LasR antagonist FC exerted no effect on *C. elegans* infected with the *lasR* mutant strain, as expected (Figure 9B). In contrast, curvularin (3 and 10 μM) significantly reduced the virulence of the *lasR* mutant with threefold greater potency than CPBA (Figure 9B). Additionally, to confirm that the protective effects of curvularin on *C. elegans* subjected to PA14 resulted from inhibition of RhlR, the effects of curvularin on the virulence of the *rhlR* mutant were investigated. Indeed, curvularin failed to protect *C. elegans* infected with the *rhlR* mutant strain, while FC protected the worms from death, as expected (Figure 9C). Taken together, these results indicated that curvularin prevented the *in vivo* virulence of the wild-type strain and *lasR* mutant in *C. elegans* by acting on RhlR.

## Discussion

Virulence factor production and biofilm formation in *P. aeruginosa* are regulated by the hierarchical QS network in which the Las system activates both the Rhl and Pqs systems (Lee and Zhang, 2015). However, recent studies have shown that the Rhl system can be activated through a Las-independent mechanism (Lee et al., 2013). Importantly, in clinical isolates of *P. aeruginosa* from CF patients, the Las system was frequently nonfunctional due to *lasR* mutations, and therefore, RhlR independently activated QS-dependent genes (Feltner et al., 2016). Additionally, it has been reported that RhlR plays a central role in mediating *in vivo* QS (O'Loughlin et al., 2013; Waters and Goldberg, 2019). Thus, RhlR antagonists are needed to prevent and treat chronic *P. aeruginosa* infections.

In this study, curvularin showed differential inhibition of virulence factor production and biofilm formation, a previously unreported outcome. At the low concentrations of 1–30 μM, curvularin potently inhibited pyocyanin and rhamnolipid production at a level similar to that of FC without affecting cell growth, but did not inhibit biofilm formation or elastase production in *P. aeruginosa* PA14 and PAO1. Elastase production and biofilm formation were negligibly inhibited by curvularin at concentrations greater than 100 μM. On the other hand, low-concentration CPBA, which has been reported to an RhlR antagonist in *E. coli* reporter strains, not only inhibited pyocyanin and rhamnolipid production but also weakly inhibited biofilm formation and elastase production in PA14 and PAO1. Considering that pyocyanin and rhamnolipid are RhlR-directed exoproducts and that biofilm formation is regulated by the Las system, these results suggested that curvularin had more selective RhlR inhibition than that exhibited by CPBA. By analyzing the antagonistic activities of curvularin on QS receptors using reporter strains, the production of QS signaling molecules, and the transcription of QS-related genes, we demonstrated that low-concentration curvularin selectively antagonized RhlR, and it very weakly antagonized LasR, but only at high concentrations. On the other hand, low-concentration CPBA antagonized not only RhlR but also LasR. Additionally, curvularin exhibited threefold greater inhibitory effects than CPBA or HG on the production of virulence factors and on RhlR. Because pyocyanin and rhamnolipid synthesis are regulated by both RhlR and PqsR, the exclusive RhlR-antagonizing activity of low-concentration curvularin was confirmed by investigating the effects of exogenous AHLs and PQS on curvularin-mediated inhibition of pyocyanin and rhamnolipid production in QS mutants. Indeed, low-concentration curvularin potently inhibited the production of pyocyanin and rhamnolipid through the selective inhibition of BHL-activated RhlR, whereas CPBA inhibited RhlR and LasR weakly. Additionally, using exogenous ligands and QS mutants, we showed that the negligible inhibition of elastase production and biofilm formation by high-concentration curvularin (more than 100 μM) was due to inhibition of OdDHL-activated LasR. Taken together, the results of this study indicate that low-concentration curvularin is an RhlR-exclusive antagonist, whereas CPBA is not an RhlR-exclusive antagonist and shows weak LasR-antagonizing activity in, at least, *P. aeruginosa*. In particular, curvularin exhibited an *in vivo* antivirulence effect by acting on RhlR with 1.25-fold higher potency than that of FC, a well-known LasR antagonist, in a *P. aeruginosa*-infected *C. elegans* model.

Previous reports have shown that elastase synthesis is activated by the *las* system and to some extent by the *rhl* system. In the *rhlI* mutant of the PAO1 strain, elastase production was reported to be partially reduced, and it was recovered by supplementation with BHL (Pearson et al., 1997). The *rhlR* mutants of the PA PG201 strain lacked elastase activity, which was restored by introducing a wild-type *rhlR* gene into the mutants (Ochsner et al., 1994; Ochsner and Reiser, 1995). However, in the present study, in the PA14 strain, low-concentration curvularin profoundly antagonized RhlR and only high-concentration curvularin (more than 100 μM) antagonized LasR and inhibited elastase, but its effects were weak (Figure 2C). Additionally, the inhibition of elastase by curvularin was reversed by adding OdDHL but not BHL (Supplementary Figure 10A). These data strongly suggest that elastase synthesis is regulated by the *las* system, not the *rhl* system, in the PA14 strain, which is inconsistent with previous reports indicating that elastase was regulated by both LasR and RhlR in *P. aeruginosa* PAO1 and PA PG201. Indeed, regulation of elastase production by the *las* system was demonstrated using QS mutants; the *lasR* mutant of the PA14 strain exhibited a remarkable reduction in elastase production compared to the wild-type strain, whereas the *rhlR* mutant showed nearly normal elastase production (Supplementary Figures 10B,C). These results strongly suggest that the regulation in elastase synthesis differs between PA14 and PAO1. Indeed, at the low concentration of 3 μM, curvularin inhibited elastase production at in PAO1, but not in PA14 (Supplementary Figure 11). The elastase inhibition by low-concentration curvularin in PAO1 was reversed by the addition of BHL, but elastase inhibition by high-concentration curvularin was reversed by supplementation with both OdDHL and BHL (Supplementary Figures 14B,C), indicating that elastase production is regulated by both LasR and RhlR in PAO1, as reported previously. Taken together, these results indicate that elastase synthesis is regulated by LasR in PA14 but by LasR and RhlR in PAO1. To the best of our knowledge, this is the first report showing that elastase synthesis in *P. aeruginosa* is differentially regulated by QS on the basis of the strain.

The relationship between QS and biofilm formation has not yet been fully determined. The Las system has been reported to play an important role in the formation of *P. aeruginosa* biofilm by regulating the expression of the *pel* or *psl* exopolysaccharide biosynthesis gene (Davies et al., 1998; Sakuragi and Kolter, 2007). Biofilm formation in the *lasI* mutant was dramatically decreased, whereas biofilm formation in the *rhlI* mutant was almost unchanged in the PAO1 strain (Davies et al., 1998). Biofilm formation in PA14 was reduced by 60% in the *lasI* and *lasR* mutant, similar to that of the *pel* mutant, whereas it is not reduced in the *rhlI* mutant and weakly reduced, by ~10%, in the *rhlR* mutant of the PA14 strain (Sakuragi and Kolter, 2007). It seems that biofilms are regulated mainly by LasR, partially by RhlR, and not regulated by RhlI. In this study, the *lasR* mutant of the PA14 strain exhibited a remarkable reduction in biofilm formation compared to the wild-type strain (Figure 8B), but the *rhlR* mutant also showed a weak reduction in biofilm formation, of ~20% (Figure 8C). These QS mutant experiments suggest partial involvement of RhlR in biofilm formation, as reported previously. However, interestingly, low-concentration curvularin did not inhibit biofilm formation but did profoundly inhibit RhlR and it very weakly inhibited biofilm formation only at a high concentration, when it very weakly inhibited LasR. These results suggest that biofilm formation is regulated by the *las* system, not the *rhl* system, in PA14. This was proven using QS mutants; the inhibition of biofilm formation by curvularin was due to inhibition of OdDHL-activated LasR, not by inhibition of RhlR (Figure 8). These results clearly demonstrate that biofilm formation is regulated by the *las* system, not the *rhl* system, in PA14. Hence, how can the possible partial involvement of RhlR in biofilm formation, as suggested by the QS mutant experiments, be explained? Considering recent reports suggesting that another RhlR pathway, which is independent of RhlI (BHL), is involved in the development of colony biofilms and the production of virulence factors (Mukherjee et al., 2017), the possibility of partial involvement of RhlI-independent RhlR in biofilm formation by PA14 is suggested; however, this question remains to be answered.

Curvularin is an aromatic compound with a cyclized alkyl side chain. It has been reported that the substitution of the homoserine lactone head group in OdDHL with an aromatic group resulted in OdDHL analogs with QS antagonism (McInnis and Blackwell, 2011; Hodgkinson et al., 2012). 6-Gingerol, a natural aromatic compound with a long alkyl side chain, was reported to show LasR antagonism by binding to LasR in a similar way to that of OdDHL (Kim et al., 2015). 4-Gingerol with a shorter side chain than that of 6-gingerol has RhlR antagonistic properties (Choi et al., 2017). Among alkyl gallates, HG, a RhlR antagonist, has the same alkyl chain length as 4-gingerol. Recent structure–activity relationship study of 4-gingerol analogs for improvement of RhlR antagonism shows that rotational rigidity between the aromatic ring and the carbonyl group of the alkyl side chain is important for enhancement of RhlR antagonism (Nam et al., 2020). Additionally, the hydroxyl moiety of the aromatic ring and the carbonyl group of the alkyl chain are important for binding to RhlR. Because a double or triple bond between the aromatic ring and the carbonyl group of the alkyl side chain increase the rotational rigidity, DFPH, a 4-gingerol analog with a triple bond, has a higher antagonism for RhlR than that of 4-gingerol analogs with a single or double bond. Considering that structurally the cyclized side chain in curvularin could restrict the rotational flexibility of the side chain more strongly than the double or triple bond, it was suggested that the higher specificity of curvularin for RhlR than that of HG or CPBA might be due to its more rotational rigidity of the cyclized side chain.

Many small-molecule inhibitors of QS have been reported to be derived from natural sources, including bacteria, cyanobacteria, fungi, algae, and plants (Zhao et al., 2019; Hemmati et al., 2020). Brominated furanone, the first natural product obtained from marine algae, competitively inhibits the LuxR receptor. Penicillic acid, patulin, equisetin, and terrein extracted from fungi inhibit both LasR and RhlR (Rasmussen et al., 2005; Kim et al., 2018). Coumarin, curcumin, berberine, cinnamic acid, and 6-gingerol extracted from plants inhibit LasR, while flavonoids inhibit both LasR and RhlR (Kim et al., 2015). All the reported natural QS inhibitors inhibit both biofilm formation and virulence factor production in *P. aeruginosa*. To the best of our knowledge, curvularin is the first RhlR antagonist isolated from natural sources.

Many synthetic QS inhibitors have been developed to block QS in *P. aeruginosa* (Galloway et al., 2011; Kalia, 2013). Most of these QS inhibitors have been focused on LasR because of its location at the top of the *P. aeruginosa* QS receptor hierarchy where it regulates *rhl-* and pqs-associated genes. Synthetic analogs of halogenated furanones including furanone C-30, or synthetic compounds of nonnatural origin such as V-06-018 and PD12, have been reported (Galloway et al., 2011). However, although RhlR plays an important role in QS in *P. aeruginosa*, RhlR antagonists have rarely been reported. Recently, Bassler et al. reported mBTL to be a partial antagonist of RhlR that inhibits pyocyanin production and prevents nematode death in a *P. aeruginosa* infection model, suggesting that RhlR may be a promising target for antivirulence treatment (O'Loughlin et al., 2013). Blackwell et al. extensively screened a focused library of synthetic, nonnative BHL analogs to use in the development of small-molecule probes that can alter the activities of individual QS receptors (Welsh et al., 2015; Boursier et al., 2018), and they thus identified CPBA and 4-iodo phenoxyacetyl L-homoserine lactone (4-iodo POHL) as RhlR antagonists in *E. coli* reporter strains. Park et al. reported an alkynyl ketone class compound, 1-(3,4-difluorophenyl)hex-1-yn-3-one (DFPH), to be a RhlR antagonist, a synthetic analog of 4-gingerol, in *E. coli* reporter strains (Nam et al., 2020). Interestingly, at low micromolar concentrations, CPBA, which has been proposed to be an RhlR antagonist, has been reported to inhibit pyocyanin and rhamnolipid production as well as biofilm formation in *P. aeruginosa* (Nam et al., 2020), exhibiting the same phenotype as CPBA in this study. However, in the present study, CPBA antagonized not only RhlR but also LasR, although weakly. CPBA inhibited pyocyanin and rhamnolipid production by antagonizing both LasR and RhlR (Figures 6B, 7A; Supplementary Figures 5A,B) and biofilm formation by antagonizing LasR but not RhlR (Figures 8A,C), as shown with the QS mutants in this study. Because DFPH has also been reported to inhibit both virulent factor production and biofilm formation at the same low concentrations (Nam et al., 2020), which reflect the same phenotype as that induced by CPBA, it was suggested that both CPBA and DFPH are not exclusive RhlR antagonists and that they retain weak LasR-antagonizing activity in *P. aeruginosa*. Taken together, the results of this study suggest that, at low micromolar concentrations, curvularin is an RhlR-exclusive antagonist, whereas CPBA is an RhlR antagonist with weak LasR-antagonizing activity in *P. aeruginosa*.

Antagonizing effects on QS receptors have generally been investigated in QS reporter strains of *E. coli*, *C. violaceum* CV026/*A. tumefaciens NT1* pair, or *P. aeruginosa*. The QS-antagonizing activity of CPBA and DFPH have been reported in *E. coli* QS reporter strains (Boursier et al., 2018; Nam et al., 2020). CPBA reportedly antagonized RhlR with a half-maximal inhibitory concentration (IC_50_) of 52.2 μM, but it did not antagonize LasR even when it was applied at 1 mM in an *E. coli* QS reporter strain (Boursier et al., 2018), which is inconsistent with the results of CPBA in *C. violaceum* CV026 and *A. tumefaciens* NT1 and in *P. aeruginosa* QS mutants in this study. These results suggest that the *E. coli* QS reporter strain might be less sensitive to LasR antagonists than *P. aeruginosa*. Indeed, 4-iodo POHL has been reported to show a similar antagonizing activity toward RhlR but a different antagonizing activity toward LasR in *E. coli* and *P. aeruginosa* reporter strains; specifically, this compound did not antagonize LasR at 100 μM in *E. coli* reporter strains but did antagonize LasR by 60% at 100 μM in *P. aeruginosa* reporter strains (Eibergen et al., 2015; Welsh et al., 2015). Taken together, these data suggest that reporter strains of *P. aeruginosa* may yield more accurate QS competition assay results than *E. coli*, especially with respect to LasR.

Under standard laboratory conditions, the Las system directs the expression of both the Rhl and Pqs systems. However, it has been reported that this regulatory hierarchy can be nutritionally and environmentally rewired. Under low-phosphate growth conditions, Rhl and Pqs are activated independently of Las (Meng et al., 2020). Depletion of phosphate, an essential component of energy molecules, such as ATP and nucleic acids, is evident after surgical injury, increasing virulence of *P. aeruginosa* (Long et al., 2008; Chekabab et al., 2014). When responding to phosphate depletion stress, the PhoRB regulator of the two-component system activates Rhl and Pqs by profoundly competing against LasR (Meng et al., 2020). Additionally, *lasR* is frequently mutated and nonfunctional in clinical isolates of *P. aeruginosa* in the CF lung environment. In contrast, RhlR independently activates some QS-dependent genes, suggesting RhlR plays a central role in the *in vivo* QS of *P. aeruginosa* during infections of patients with CF (Bjarnsholt et al., 2010; Feltner et al., 2016). Kostylev et al. (2019) and Oshri et al. (2018) also suggested a primary mechanism by which RhlR can be activated when LasR is nonfunctional under laboratory conditions. However, more information on QS circuits under clinical *lasR*-nonfunctional conditions is needed; what are the connections between RhlR and the PQS system, what genes are regulated by RhlR, do RhlR-regulated genes depend on BHL? Because low micromolar concentrations curvularin is a potent and exclusive inhibitor of BHL-mediated activation of RhlR, curvularin can be useful for the development of novel agents to treat *P. aeruginosa* infections as well as for elucidation of the RhlR-mediated QS circuits in *P. aeruginosa.*

(−)-Curvularin has been reported to be produced by many endophytic and soil fungal species, such as *Penicillium* spp. (Yao et al., 2003), *Aspergillus* spp. (Caputo and Viola, 1977), *Curvularia* spp. (Greve et al., 2008), *Alternaria* spp. (Robeson et al., 1985), and *Epicoccum* spp. (Abdel-Hafez et al., 2017). This is the first study to isolate curvularin from *Phoma* sp. Curvularin was isolated as a white powder in this study and soluble in methanol, ethanol, and acetone. Curvularin has no antimicrobial activity including Gram-positive and -negative bacteria and *Candida albicans* (Kumar et al., 2013). The compound is weak cytotoxic or inactive against human tumor cell lines whereas some curvularin analogs including α,β-dehydrocurvularin show potent cytotoxicity (Kumar et al., 2013; Meng et al., 2013), but is nontoxic to mice and chick embryo (Vesonder et al., 1976). Curvularin exhibits anti-inflammatory activities by inhibiting human inducible nitric oxide synthase (iNOS) expression and iNOS-dependent NO production (Schmidt et al., 2010). However, the antivirulence activity of curvularin against bacteria had not been previously reported.

## Conclusion

Curvularin potently inhibited pyocyanin and rhamnolipid production, showing activity similar to that of FC, by antagonizing only RhlR when administered at the low micromolar concentrations of 1–30 μM, without affecting the cell viability of *P. aeruginosa*. Furthermore, curvularin showed threefold greater inhibition of virulent factor production and more selective antagonism against RhlR than CPBA, which retained a weak LasR-antagonizing activity in *P. aeruginosa*, and hexyl gallate. Additionally, the QS mutant-based investigation into the inhibitory relationship between QS and virulent factors by low-concentration curvularin indicated that curvularin inhibited pyocyanin and rhamnolipid production by selectively antagonizing RhlI-dependent RhlR. In particular, curvularin exhibited an *in vivo* antivirulence effect by acting on RhlR with 1.7-fold greater potency than that of FC in a *P. aeruginosa*-infected *C. elegans* model. Overall, low-concentration curvularin is an RhlR-exclusive antagonist in *P. aeruginosa*, and to the best of our knowledge, this is the first report describing an RhlR antagonist from natural resources. Hence, curvularin may be useful for developing novel agents that inhibit the LasR-independent Rhl QS system, which functions in chronic *P. aeruginosa* infections, and for studying the role of RhlR in complex QS networks.

## Data Availability Statement

The datasets presented in this study can be found in online repositories. The names of the repository/repositories and accession number(s) can be found at: figshare, https://figshare.com/s/9111198a877d87c2a642.

## Author Contributions

W-GK conceived and designed the experiments and wrote the manuscript. H-YC performed most of the experiments. DL identified the structure of curvularin. H-YC and W-GK analyzed the experimental data. All authors contributed to the article and approved the submitted version.

## Funding

This work was supported by Basic Science Research Program through the National Research Foundation of Korea (NRF) funded by the Ministry of Education (grant number NRF-2021R1I1A2048905) and the Korea Research Institute of Bioscience & Biotechnology (KRIBB) Research Initiative Program, South Korea.

## Conflict of Interest

The authors declare that the research was conducted in the absence of any commercial or financial relationships that could be construed as a potential conflict of interest.

## Publisher’s Note

All claims expressed in this article are solely those of the authors and do not necessarily represent those of their affiliated organizations, or those of the publisher, the editors and the reviewers. Any product that may be evaluated in this article, or claim that may be made by its manufacturer, is not guaranteed or endorsed by the publisher.
