# Supplementary material for: Curvularin Isolated From Phoma macrostoma Is an Antagonist of RhlR Quorum Sensing in Pseudomonas aeruginosa
Source: Front Microbiol. 2022 Jul 12;13:913882. doi: 10.3389/fmicb.2022.913882 (PMC9315252; doi:10.3389/fmicb.2022.913882)
Supplement: Supplementary file 1 [file Data_Sheet_1.pdf]

Supplementary Material

Curvularin Isolated from *Phoma macrostoma* is an Antagonist of RhlR Quorum Sensing in *Pseudomonas aeruginosa*

Ha-Young Choi[1,2], Duc Dat Le[1], Won-Gon Kim [1,2,#]

[1]Infectious Disease Research Center, Korea Research Institute of Bioscience and Biotechnology, Yusong, Daejeon, Korea.

[2]Department of Bio-Molecular Science, KRIBB School of Bioscience, Korea University of Science and Technology (UST), Yusong, Daejeon, Republic of Korea.

Running Head: a new RhlR antagonist against *P. aeruginosa* infection

# Address correspondence to Won-Gon Kim, wgkim@kribb.re.kr

Quantification of QS signaling molecules by LC-MS/MS

The samples were subjected to an HPLC system (Luna C18(2), 100 × 2.0 mm, 3 μm, Phenomenex, Torrance, CA, USA) connected to a QTrap 3200 with a TurboIon Spray source (AB SCIEX, Singapore). The column was maintained at 20°C with a flow rate of 0.4 mL/min and a gradient of acetonitrile in 0.1% (v/v) aqueous formic acid; 0-10 min from 70% to 100%. MRM was performed by selecting the two mass ions set specifically for the selected analytes to detect the transition from parent ion to product ion, i.e., *m/z* 298.211 > 197.200 for OdDHL, *m/z* 172.181 > 71.000 for BHL, and *m/z* 260.244 > 188.100 for PQS (Sigma). For analysis of OdDHL, BHL, and PQS, the TurboIon Spray source-dependent parameters were optimized to the following values: 10 psi curtain gas, high collision gas, 5500 V ion spray voltage, 400°C temperature, and 12 psi ion source gas. The compound-dependent parameters for OdDHL, BHL, and PQS were optimized to the following values: 19, 17, and 41 eV collision energy; 5, 8, and 12 V entrance potential; 276, 31, and 71 V declustering potential; and 18, 10, and 16 V collision cell exit potential, respectively.

RT-qPCR of QS-regulated genes

cDNA was synthesized from 2 μg of RNA mixed with 1 μg of random primers (Promega C1181) and RNase-free water (Sigma W4502) in a total of 13.37 μL and incubated for 5 min at 70°C. Thereafter, 1 μL of M-MLV reverse transcriptase (Promega M170 200 U/μL), 5 μL 5 × M-MLV buffer (Promega M531), 5 μL of deoxynucleoside triphosphates (Enzynomics N001S, 2 mM), and 0.63 μL of RNasin® Ribonuclease Inhibitors (Promega N251, 2500 U/μL) were added and incubated for 60 min at 42°C. The cDNA samples were used for RT-qPCR detection of the expression of target genes. RT-qPCR was performed using the Bio-Rad CFX-96 real time system (Bio-Rad, Hercules, CA, USA) with the primers listed in Table S1. Amplification and expression were carried out in a total volume of 20 μL containing 10 μL SYBR Premix Ex Taq™ (Takara, Shiga, Japan), 1 μL each of the forward and reverse primers (5 μM) of target

genes, 2 μL template cDNA, and 6 μL RNase-free water. The cycling parameters were as follows: initial activation at 95℃ for 30 s; 40 cycles at 95℃ for 5 s, 60℃ for 30 s, and melting curve analysis at 95℃ for 15 s, 60℃ for 5 s and 95℃ for 5 s. mRNA expression was normalized using the endogenous *rpoD* gene.

Supplementary Table 1. Primers used for quantitative RT-PCR

Gene and primer type	PCR primer sequence (5' to 3')
lasI	
Forward	TTCAAGGAGCGCAAAGGCTG
Reverse	GTTCTTCAGCATGTAGGGGC
lasR	
Forward	TCTGGGAACCGTCCATCTAC
Reverse	GACCGACTCCATGAAACGGT
lasA	
Forward	GACGACCTGTTCCTCTACGG
Reverse	GCTCCAGGTATTCGCTCTTG
lasB	
Forward	CCGCAAGACCGAGAATGACA
Reverse	CTTCCCACTGATCGAGCACT
aprE	
Forward	ATGTACATCGTGCCCAACAG
Reverse	GGTCTTGCTCTGGTTGAAGG
rhlI	
Forward	CTTCATCGAGAAGCTGGGCT
Reverse	AGGTAGGCGAAGACGTCCTT
rhlR	
Forward	TGCATGATCGAGTTGCTG
Reverse	GTGCTCTCGGAGATGCTCA
rhlA	
Forward	GCGCGAAAGTCTGTTGGTAT
Reverse	CAGCACCACGTTGAAATGTT
pqsA	
Forward	CCACTCCGCTGGACGACAAC
Reverse	GCAGCATGTGCGAGGGAATC
phnB	
Forward	CACTCGCTGGTGGTCAGTC
Reverse	AGAGTAGAGCGTTCTCCAGCA
pqsH	
Forward	ATGTCTACGCGACCCTGAAG
Reverse	AACTCCTCGAGGTCGTTGTG
pqsR	
Forward	CTTCGCCTGATCCCTTACAT
Reverse	TGAAATCGTCGAGCAGTACG
phzA2	
Forward	AACCACTTCTGGGTCGAGTG
Reverse	TCGAGTTCGAAGGAATGGAT
rpoD	
Forward	GGGGATCAACGTATTCGAGA
Reverse	GGTACCCATTTCACGCATGT

Supplementary Table 2. ^{1}H and ^{13}C NMR data for the isolated curvularin ($CDCl_3$, 500 MHz).

Position	δ_H (m[a], *J* in Hz)	δ_C
1	-	171.9
2	3.67 (1H, d, 15.7)<br>3.72 (1H, d, 15.7)	40.1
3	-	135.8
4	6.19 (1H, brs)	112.1
5	-	159.8
6	6.19 (1H, brs)	102.3
7	-	159.6
8	-	119.6
9	-	208.8
10	2.77 (1H, m)<br>3.05 (1H, ddd, 3.8, 8.2, 14.8)	43.3
11	1.58 (1H, m)<br>1.68 (1H, m)	22.8
12	1.24 (1H, m)<br>1.38 (1H, m)	26.9
13	1.22 (1H, m)<br>1.37 (1H, m)	23.2
14	1.42 (1H, m)<br>1.55 (1H, m)	31.9
15	4.88 (1H, m)	73
15-CH_3	1.09 (3H, d, 6.3)	20

[a] multiplicity

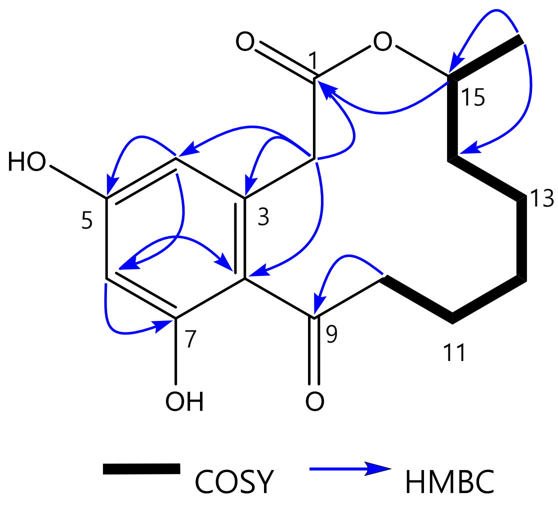

Supplementary Figure 1. ^{1}H-^{1}H COSY and HMBC correlations of curvularin.

.

A

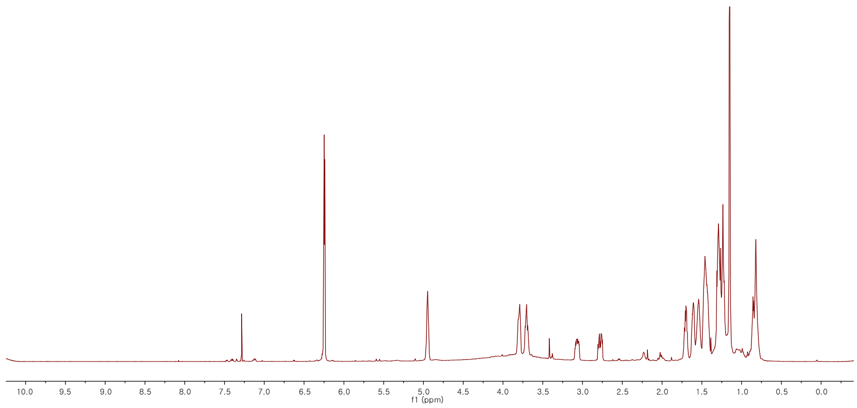

10.0
9.5
9.0
8.5
8.0
7.5
7.0
6.5
6.0
5.5
5.0
4.5
4.0
3.5
3.0
2.5
2.0
1.5
1.0
0.5
0.0
f1 (ppm)

B

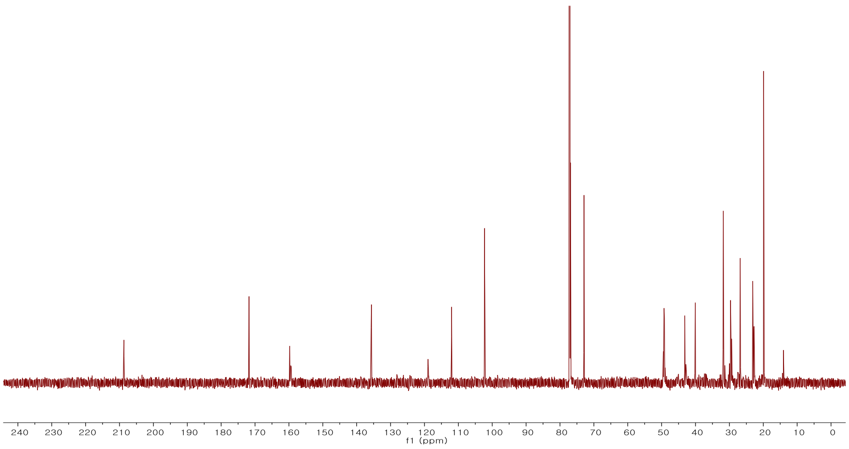

240
230
220
210
200
190
180
170
160
150
140
130
120
110
100
90
80
70
60
50
40
30
20
10
0
f1 (ppm)

C

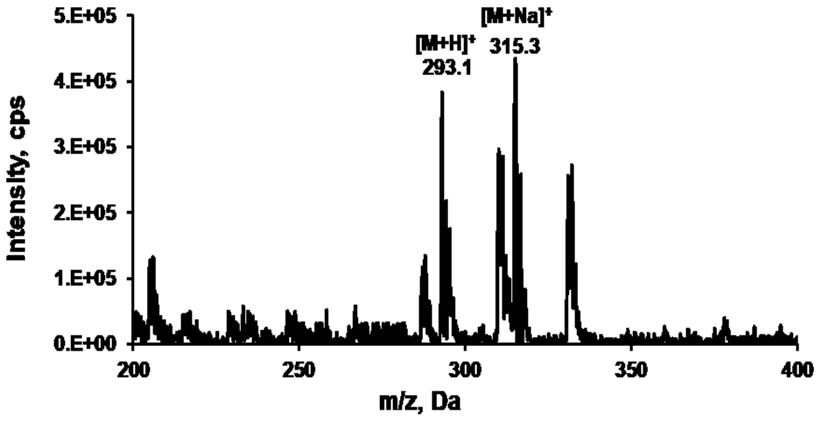

5.E+05
4.E+05
3.E+05
2.E+05
1.E+05
0.E+00
Intensity, cps
[M+H]+
293.1
[M+Na]+
315.3
200
250
300
350
400
m/z, Da

Supplementary Figure 2. ^{1}H (**A**), ^{13}C NMR (**B**), and ESI-MS (**C**) spectra of the isolated curvularin. NMR was measured in $CDCl_3$ at 500MHz.

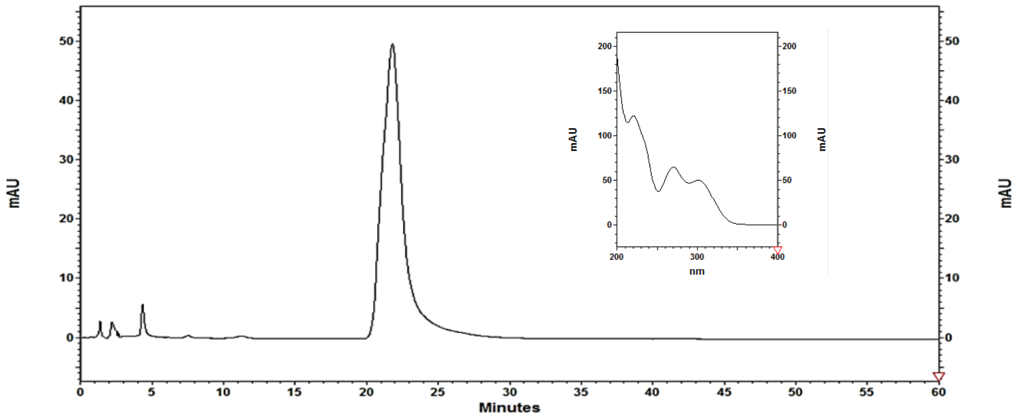

Supplementary Figure 3. HPLC profile of curvularin isolated from *Phoma macrostoma* FN413. The purity of the compound was analyzed at 220 nm by analytical HPLC column (J′sphere ODS-H80, 150 x 4.6 mm I.D., YMC) chromatography with acetonitrile:water (30:70) containing 0.01 % trifluoroacetic acid at a flow rate of 0.8 ml/minute.

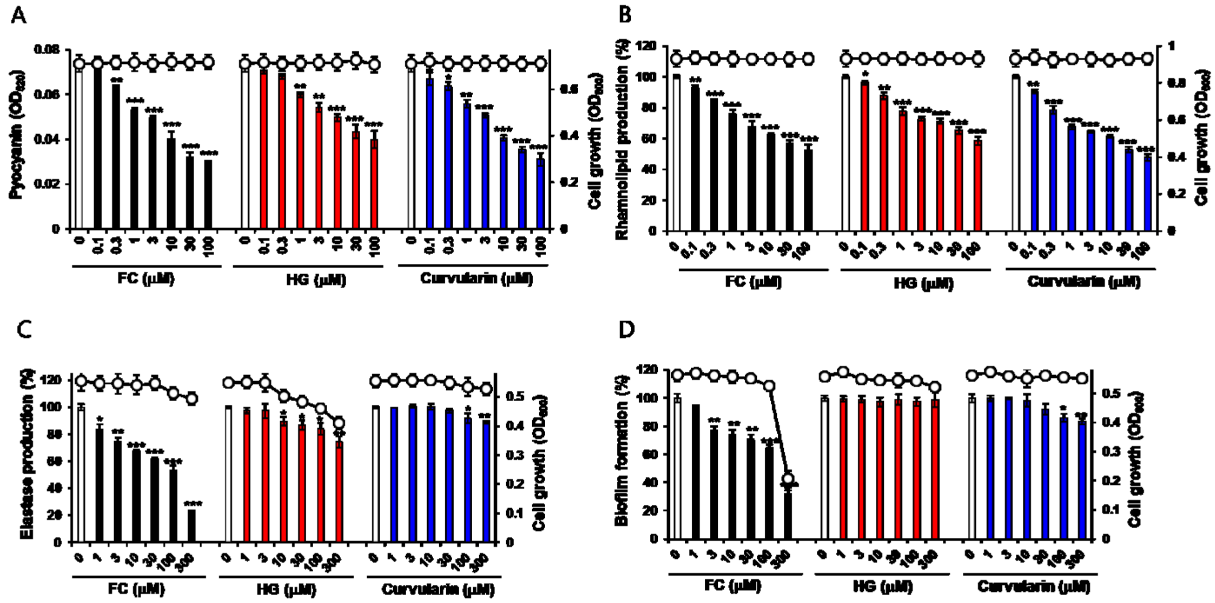

Supplementary Figure 4. Comparison of the effects of curvularin and hexyl gallate on *P. aeruginosa* virulence factor production and biofilm formation. (**A, B, C**) Effects of curvularin on virulence factor production and cell viability in *P. aeruginosa* PA14. After PA14 cells were grown in LB medium containing various concentrations of curvularin for 24 h, cell density was measured at 600 nm and pyocyanin and rhamnolipid and elastase activity in the culture supernatants were then determined. **(D)** Effects of curvularin on *P. aeruginosa* biofilm formation and cell viability. PA14 biofilms were grown in the presence of curvularin for 9 h, followed by the measurement of planktonic cell density at 600 nm. The biofilm cells attached to the well surface were assayed using crystal violet staining. Furanone C-30 (FC) and hexyl gallate (HG) are known antagonists of LasR and RhlR, respectively. Three independent experiments were performed in triplicate, and the mean ± SD values are presented in each bar. * $P < 0.01$; **, $P < 0.001$; and $^{\#}$, $P < 0.0001$ versus untreated cells.

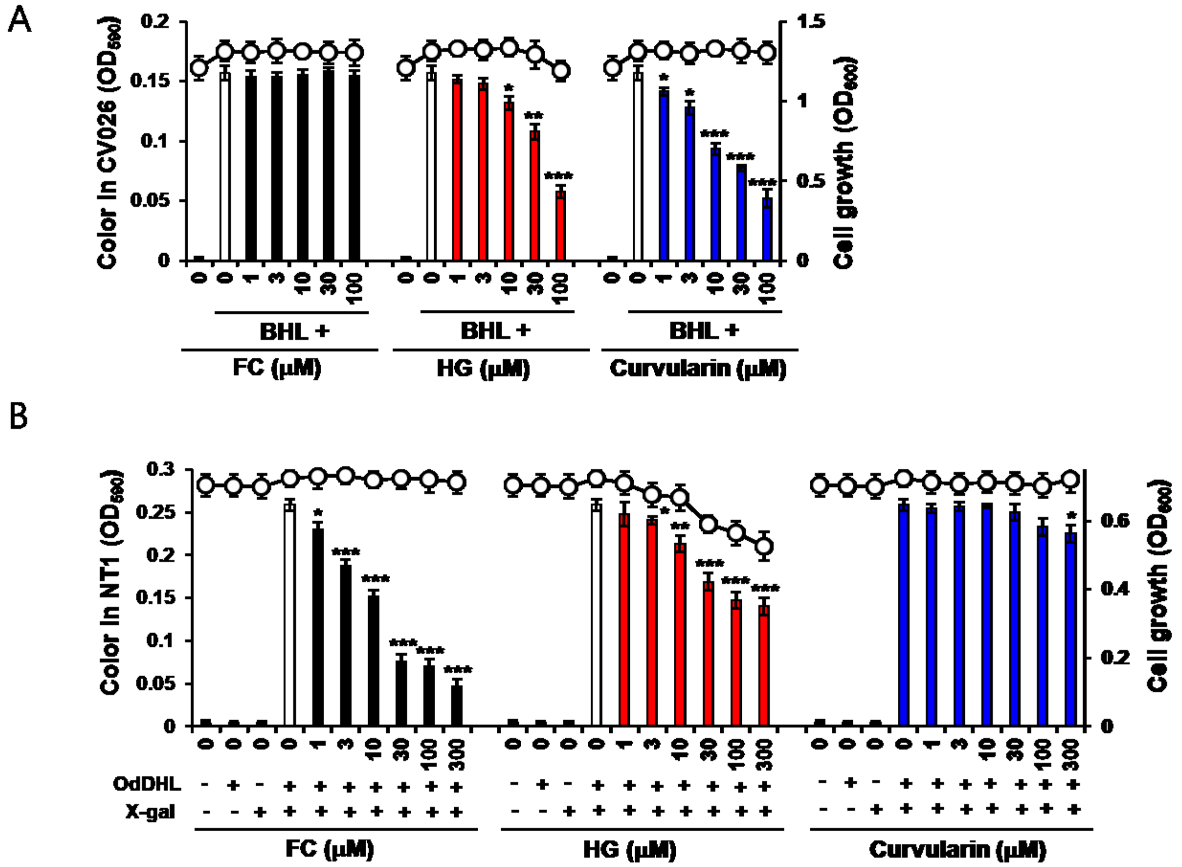

Supplementary Figure 5. Comparison of the effects of curvularin and hexyl gallate on QS. QS competition against BHL and OdDHL was performed using the reporter strains *A. tumefaciens* NT1 and *C. violaceum* CV026, respectively. After 1 μM OdDHL and 500 μM BHL were added to the NT1 and CV026 cultures, respectively, with various concentrations of curvularin and incubated for 24 h, cell growth at 600 nm and color change intensity were measured. (**A**) Curvularin – BHL competition. (**B**) Curvularin – OdDHL competition. Furanone C-30 (FC) and hexyl gallate (HG) are known antagonists of LasR and RhlR, respectively. The data are expressed as the mean ± SD values of three independent experiments performed in triplicate. *, $P < 0.01$; **, $P < 0.001$; and $^{\#}$, $P < 0.0001$ versus untreated cells.

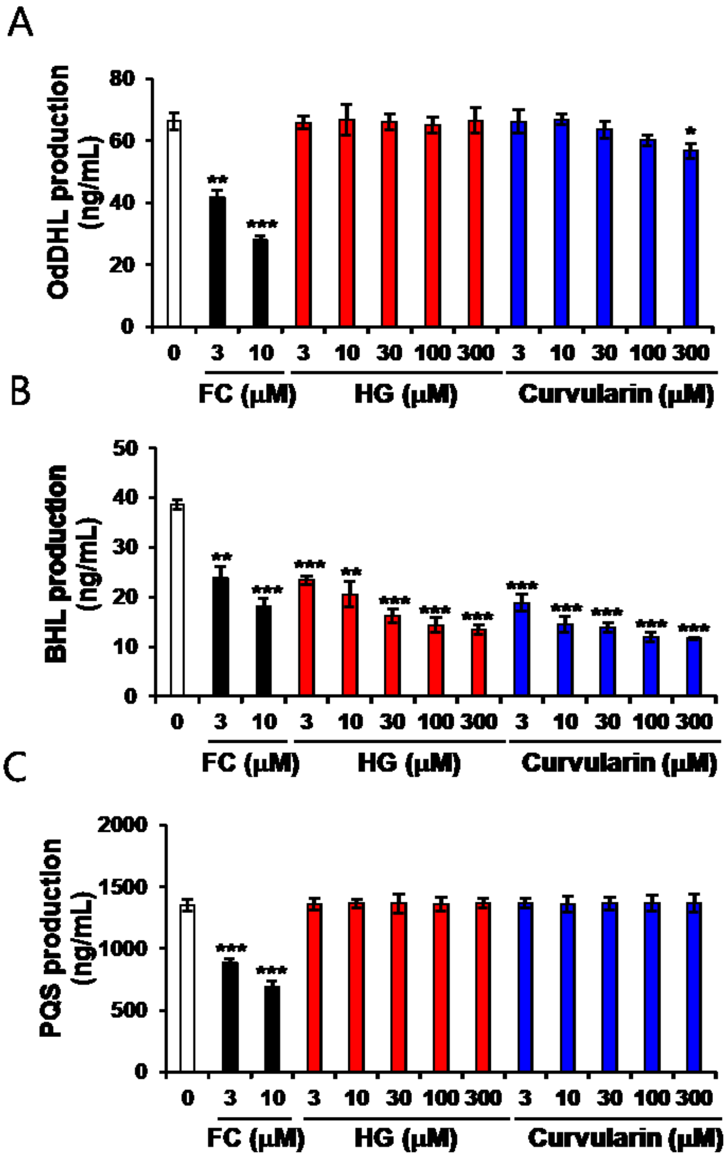

Supplementary Figure 6. Comparison of the effects of curvularin and hexyl gallate on QS signaling molecule production. PA14 cells were cultured in LB medium in the presence of curvularin for 12 h. The three main QS molecules, namely, OdDHL (**A**), BHL (**B**), and PQS (**C**), were extracted from the culture supernatants and quantitatively analyzed by LC–MS/MS. Furanone C-30 (FC) and hexyl gallate (HG) are known antagonists of LasR and RhlR, respectively. Three independent experiments were performed in triplicate, and the mean ± SD values are presented in each bar. *, $P < 0.01$; **, $P < 0.001$; and $^{\#}$, $P < 0.0001$ versus DMSO treatment.

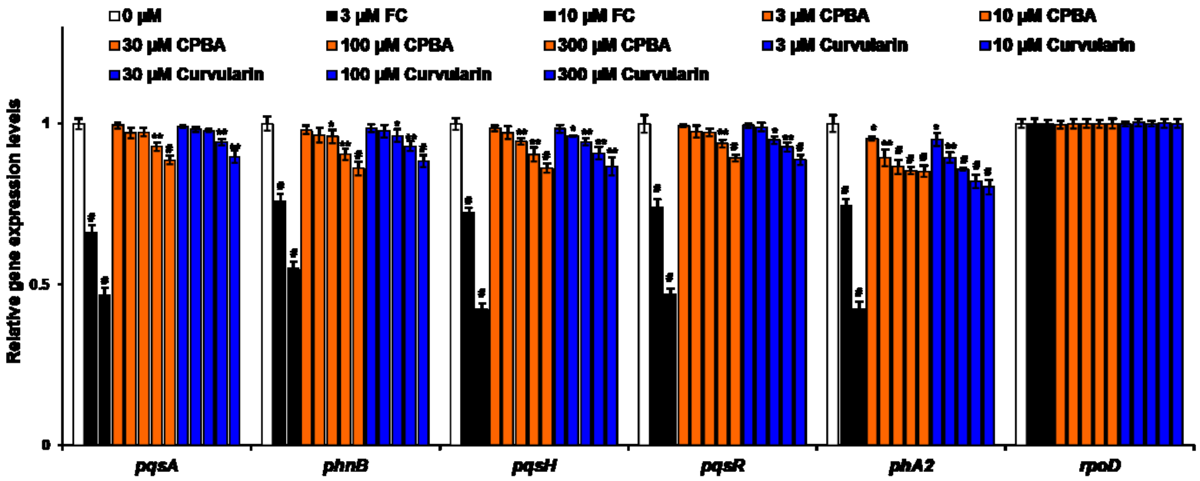

Supplementary Figure 7. Effects of curvularin on QS gene expression. PA14 cells were cultured in LB medium containing different concentrations of curvularin for 12 h. The effects of curvularin on QS gene expression were measured by RT−qPCR. Furanone C-30 (FC) and N-cyclopentylbutyramide (CPBA) are known antagonists of LasR and RhlR, respectively The experiment shown is representative of three independent experiments performed in triplicate and the mean ± SD values are presented in each bar. *, $P < 0.05$; **, $P < 0.01$; and $^{\#}$, $P < 0.001$ versus DMSO treatment.

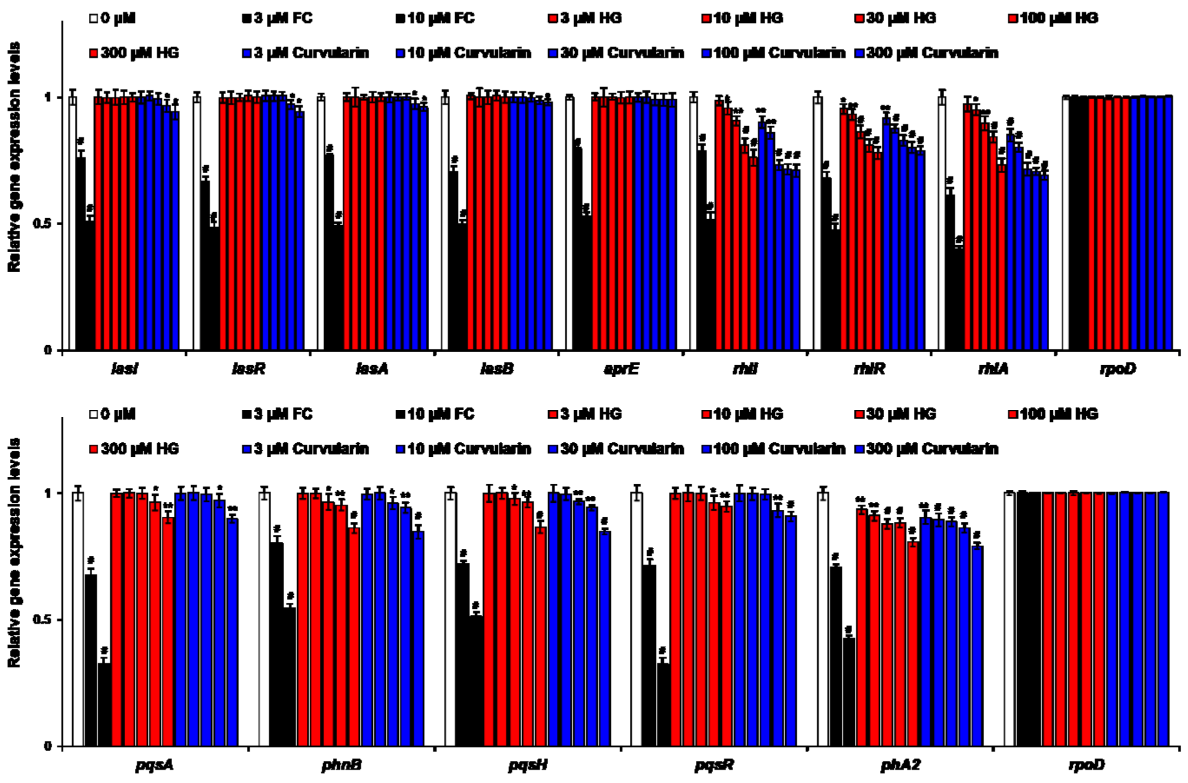

Supplementary Figure 8. Comparison of the effects of curvularin and hexyl gallate on QS gene expression. PA14 cells were cultured in LB medium containing different concentrations of curvularin for 12 h. The effects of curvularin on QS gene expression were measured by RT−qPCR. Furanone C-30 (FC) and hexyl gallate (HG) are known antagonists of LasR and RhlR, respectively. The experiment shown is representative of three independent experiments performed in triplicate and the mean ± SD values are presented in each bar. *, $P < 0.05$; **, $P < 0.01$; and #, $P < 0.001$ versus DMSO treatment.

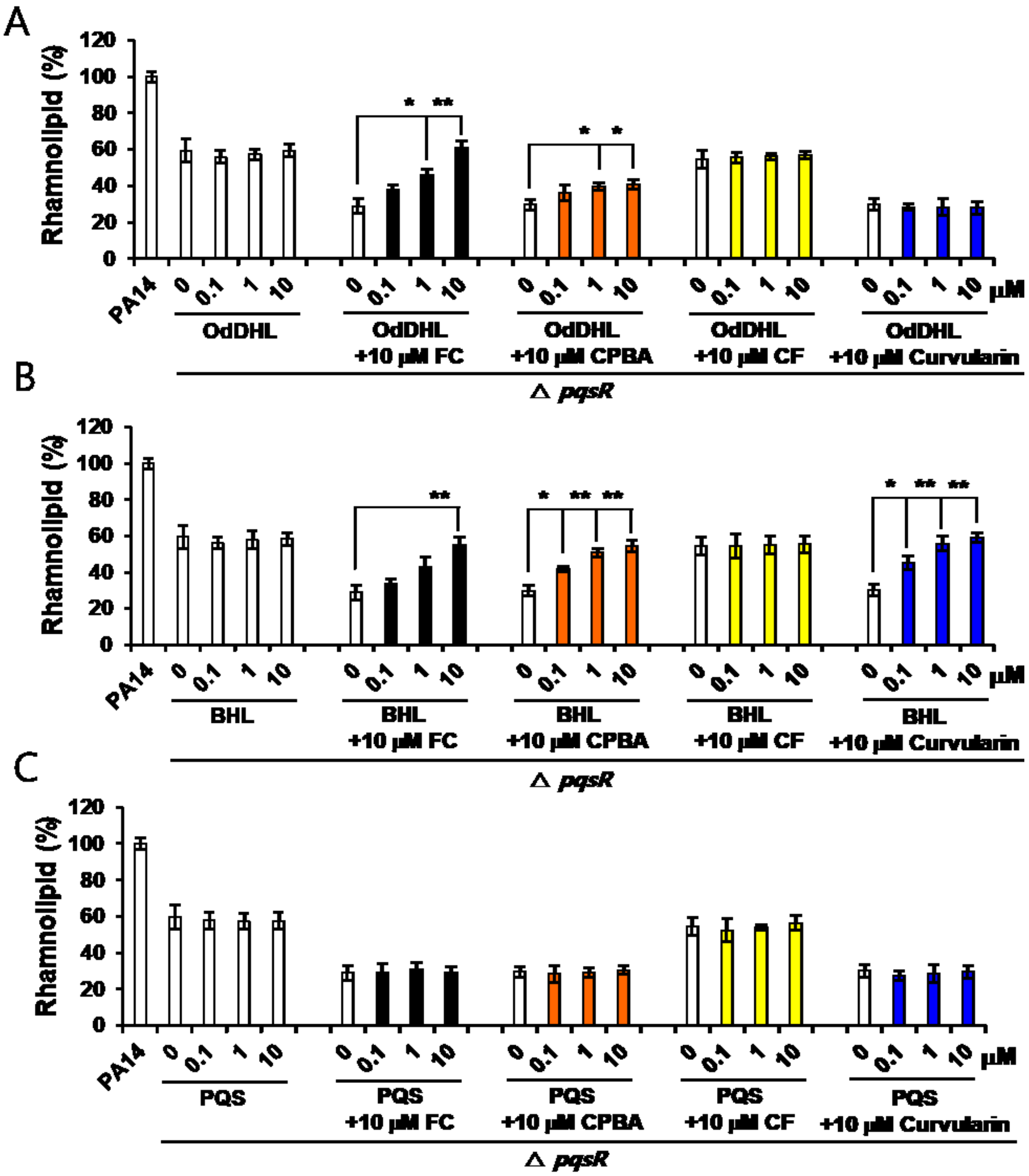

Supplementary Figure 9. Effects of exogenous QS ligands on curvularin-mediated inhibition of rhamnolipid production in the Δ*pqsR* mutant. Rhamnolipid production in the Δ*pqsR* mutant cultured with curvularin (10 μM) in the presence or absence of different concentrations of OdDHL (**A**), BHL (**B**), or PQS (**C**) for 18 h. Furanone C-30 (FC), N-cyclopentylbutyramide (CPBA), and clofoctol (CF) are known antagonists of LasR, RhlR, and PqsR, respectively. The data are representative of three independent experiments performed in triplicate and expressed as the mean ± SD values in each bar. *, $P < 0.01$; **, $P < 0.001$; and ***, $P < 0.0001$ versus DMSO treatment

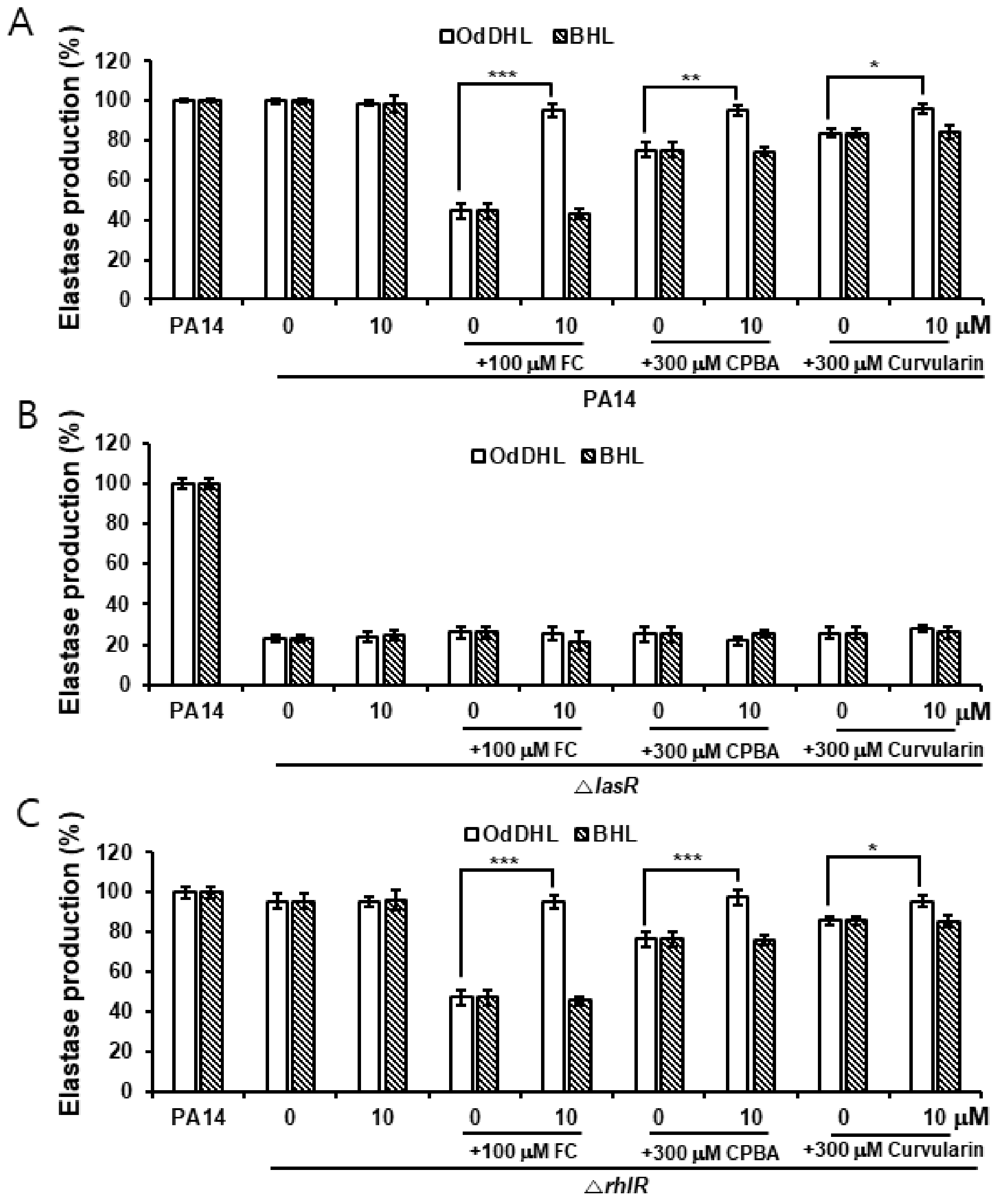

Supplementary Figure 10. Effects of exogenous QS ligands on curvularin-mediated inhibition of elastase production in the Δ*lasR* and Δ*rhlR* mutants. Elastase production in PA14 (**A**), Δ*lasR* mutant (**B**), or Δ*rhlR* mutant (**C**) cultured with curvularin (300 μM) in the presence or absence of different concentrations of OdDHL or BHL for 18 h. Furanone C-30 (FC), and N-cyclopentylbutyramide (CPBA) are known antagonists of LasR, RhlR, and PqsR, respectively. The data are representative of three independent experiments performed in triplicate and expressed as the mean ± SD values in each bar. *, $P < 0.01$; **, $P < 0.001$; and ***, $P < 0.0001$ versus DMSO treatment.

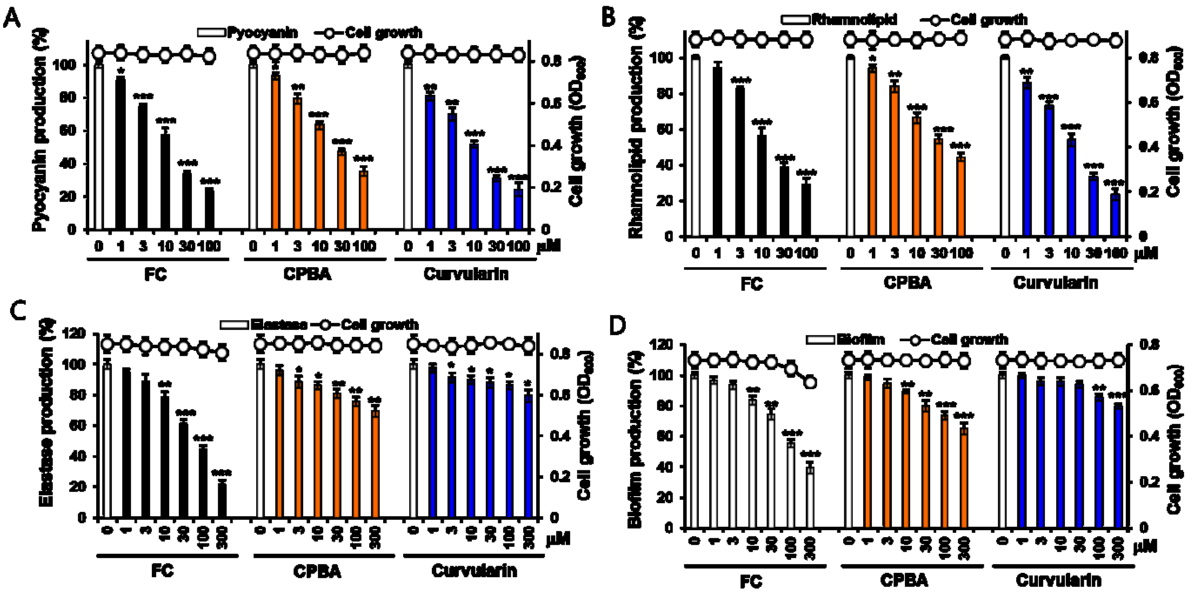

Supplementary Figure 11. Effects of curvularin on *P. aeruginosa* virulence factor production and biofilm formation in *P. aeruginosa* PAO1. (**A, B, C**) Effects of curvularin on virulence factor production and cell viability in *P. aeruginosa* PAO1. After PAO1 cells were grown in LB medium containing various concentrations of curvularin for 24 h, cell density was measured at 600 nm and pyocyanin and rhamnolipid and elastase activity in the culture supernatants were then determined. **(D)** Effects of curvularin on *P. aeruginosa* biofilm formation and cell viability. PAO1 biofilms were grown in the presence of curvularin for 9 h, followed by the measurement of planktonic cell density at 600 nm. The biofilm cells attached to the well surface were assayed using crystal violet staining. Furanone C-30 (FC) and N-cyclopentylbutyramide (CPBA) are known antagonists of LasR and RhlR, respectively. Three independent experiments were performed in triplicate, and the mean ± SD values are presented in each bar. * $P < 0.01$; **, $P < 0.001$; and $^{\#}$, $P < 0.0001$ versus untreated cells.

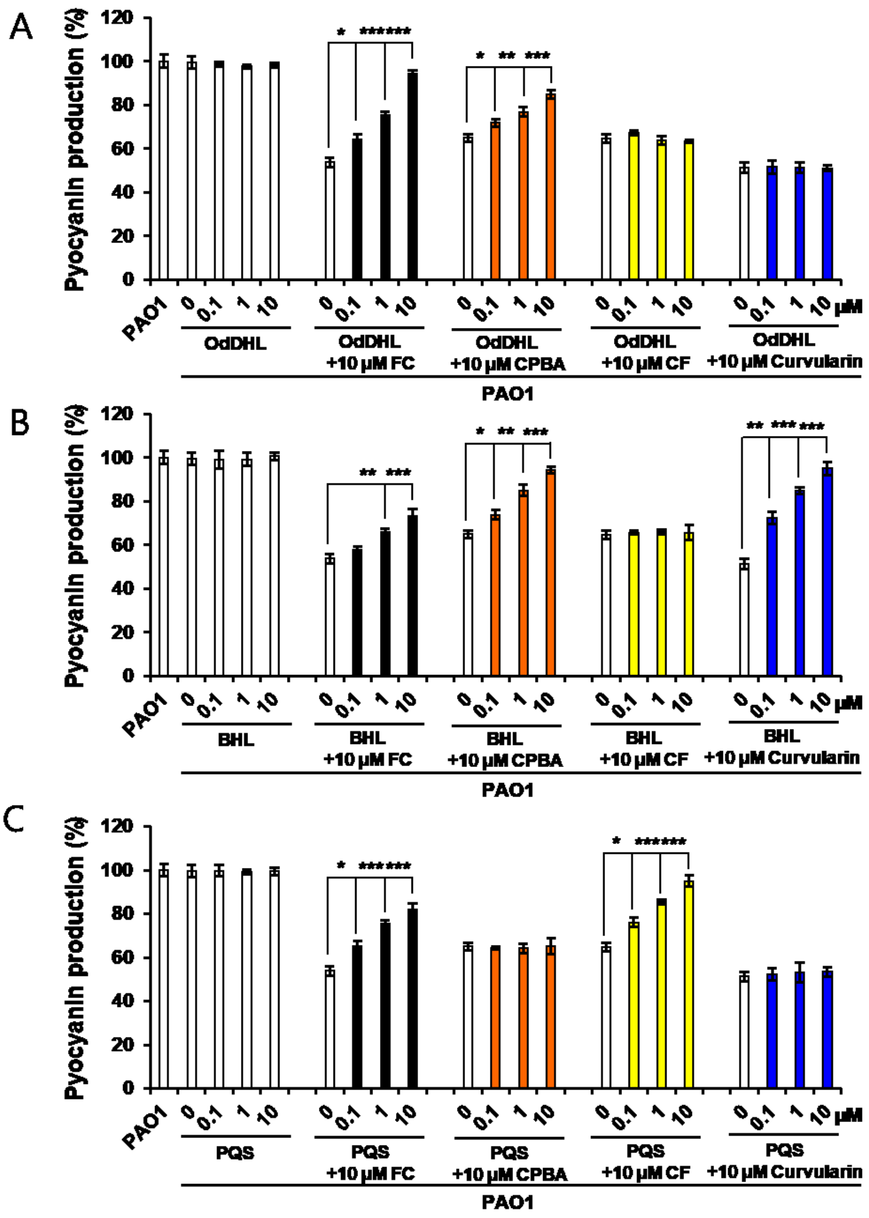

Supplementary Figure 12. Effects of exogenous QS ligands on curvularin–induced inhibition of pyocyanin production in *P. aeruginosa* PAO1. Pyocyanin production in PAO1 cultured with low-concentrations curvularin in the presence or absence of different concentrations of OdDHL (**A**), BHL (**B**), or PQS (**C**) for 18 h. Furanone C-30 (FC), N-cyclopentylbutyramide (CPBA), and clofoctol (CF) are known antagonists of LasR, RhlR, and PqsR, respectively. The data are representative of three independent experiments performed in triplicate and expressed as the mean ± SD values in each bar. *, $P < 0.01$; **, $P < 0.001$; and ***, $P < 0.0001$ versus DMSO treatment.

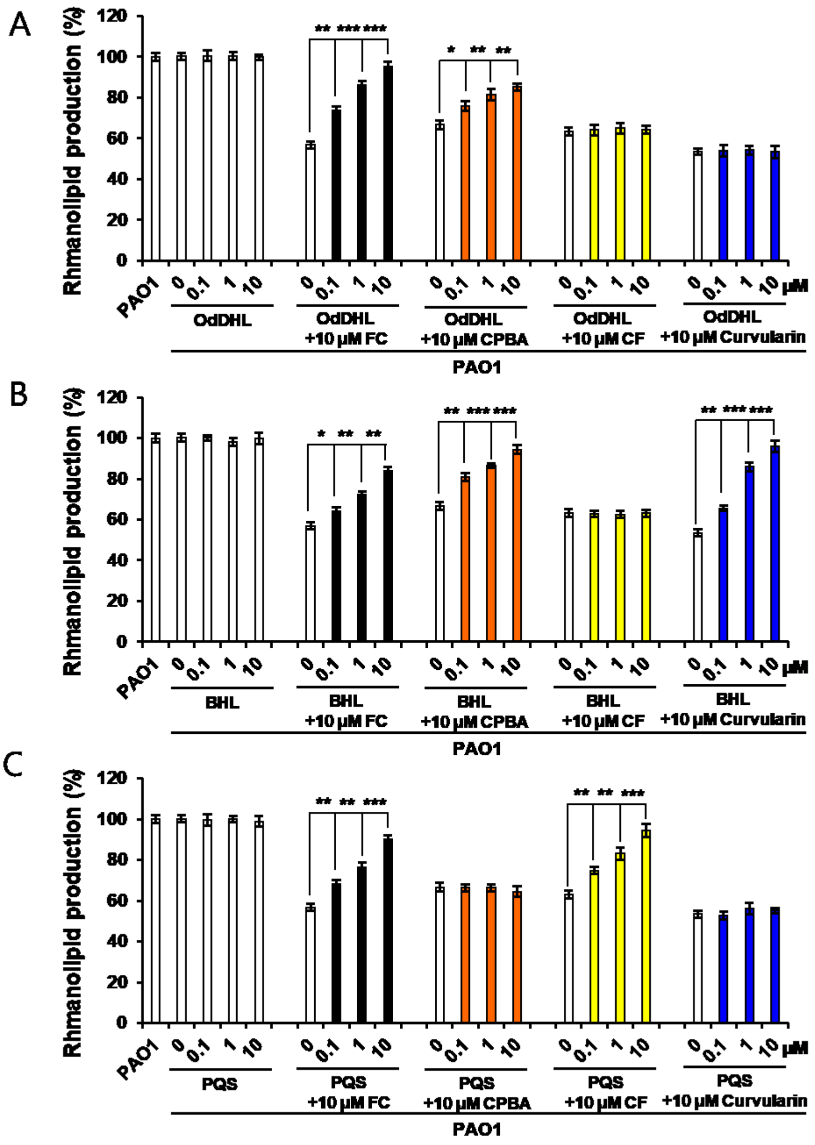

Supplementary Figure 13. Effects of exogenous QS ligands on curvularin–induced inhibition of rhamnolipid production in *P. aeruginosa* PAO1. Rhamnolipid production in PAO1 cultured with low-concentrations curvularin in the presence or absence of different concentrations of OdDHL (**A**), BHL (**B**), or PQS (**C**) for 18 h. Furanone C-30 (FC), N-cyclopentylbutyramide (CPBA), and clofoctol (CF) are known antagonists of LasR, RhlR, and PqsR, respectively.The data are representative of three independent experiments performed in triplicate and expressed as the mean ± SD values in each bar. *, $P < 0.01$; **, $P < 0.001$; and ***, $P < 0.0001$ versus DMSO treatment.

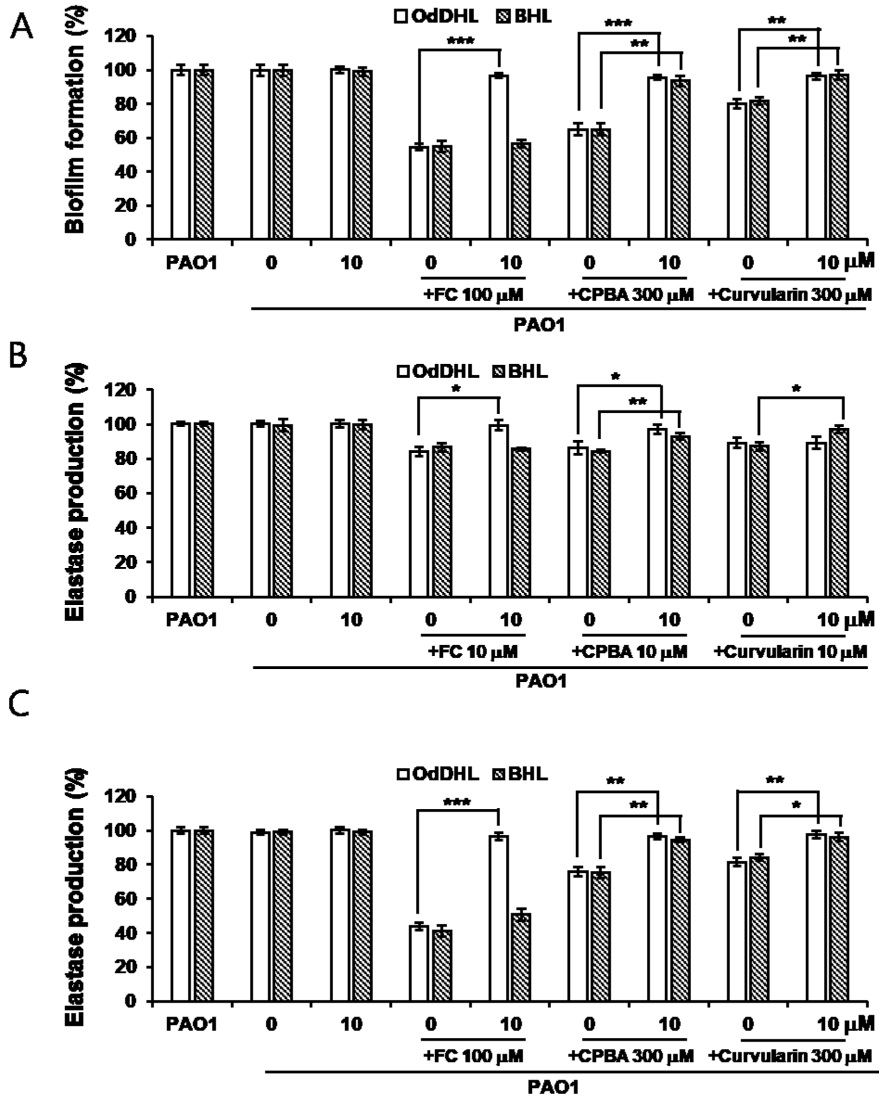

Supplementary Figure 14. Effects of exogenous QS ligands on curvularin-mediated inhibition of biofilm formation and elastase production in *P. aeruginosa* PAO1. Biofilm formation (A) and elastase production (B, C) in PAO1 cultured with low and high concentrations of curvularin in the presence or absence of different concentrations of OdDHL or BHL for 18 h. Furanone C-30 (FC) and N-cyclopentylbutyramide (CPBA) are known antagonists of LasR and RhlR, respectively. The data are representative of three independent experiments performed in triplicate and expressed as the mean ± SD values in each bar. *, $P < 0.01$; **, $P < 0.001$; and ***, $P < 0.0001$ versus DMSO treatment.

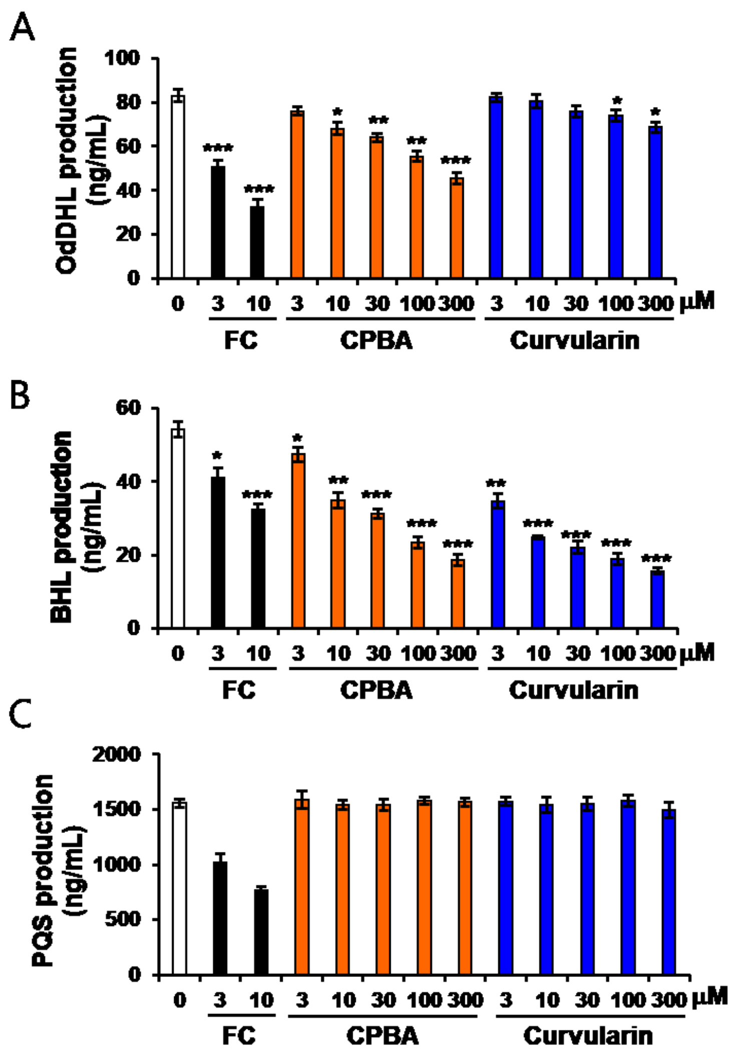

Supplementary Figure 15. Effects of curvularin on QS signaling molecule production in in *P. aeruginosa* PAO1. PAO1cells were cultured in LB medium in the presence of curvularin for 12 h. The three main QS molecules, namely, OdDHL (**A**), BHL (**B**), and PQS (**C**), were extracted from the culture supernatants and quantitatively analyzed by LC–MS/MS. Furanone C-30 (FC) and N-cyclopentylbutyramide (CPBA) are known antagonists of LasR and RhlR, respectively. Three independent experiments were performed in triplicate, and the mean ± SD values are presented in each bar. *, $P < 0.01$; **, $P < 0.001$; and $^{\#}$, $P < 0.0001$ versus DMSO treatment.

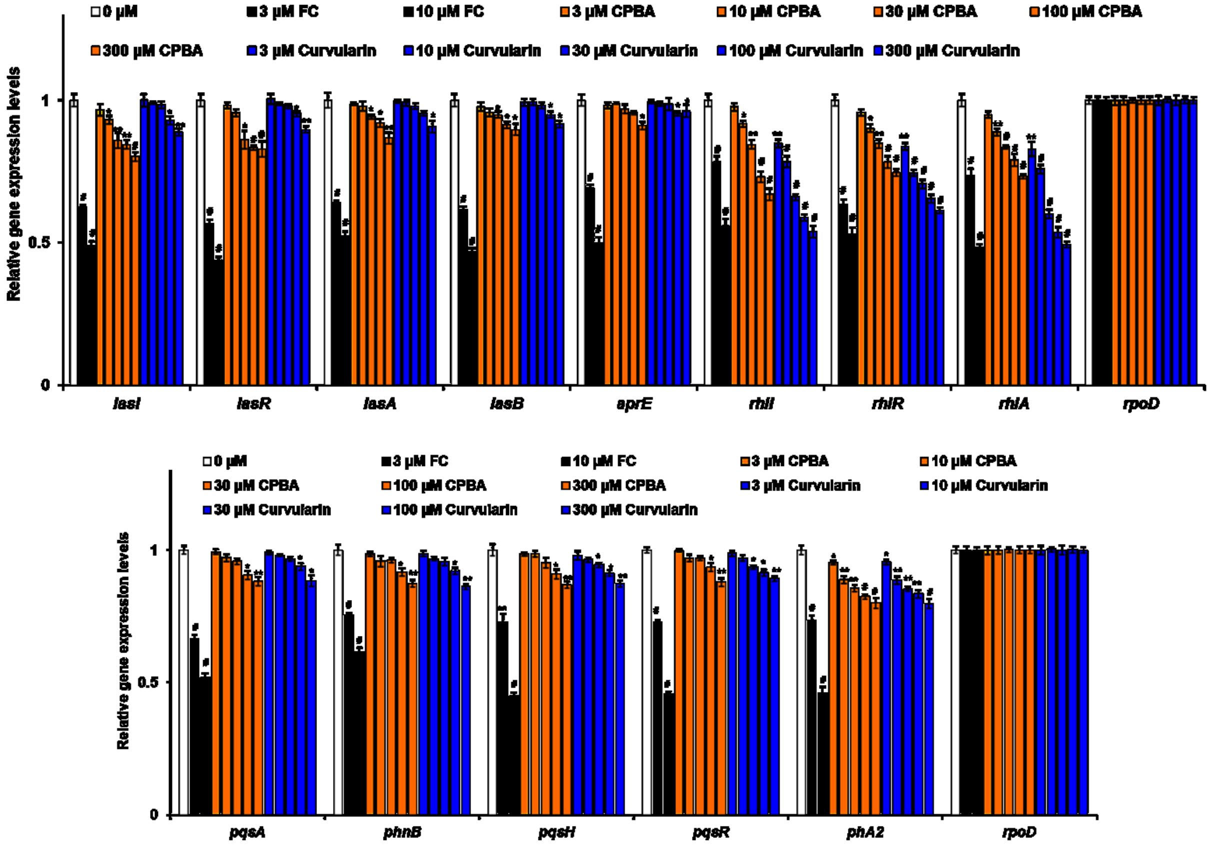

Supplementary Figure 16. Effects of curvularin on QS gene expression in *P. aeruginosa* PAO1. PAO1 cells were cultured in LB medium containing different concentrations of curvularin for 12 h. The effects of curvularin on QS gene expression were measured by RT−qPCR. Furanone C-30 (FC) and N-cyclopentylbutyramide (CPBA) are known antagonists of LasR and RhlR, respectively. The experiment shown is representative of three independent experiments performed in triplicate and the mean ± SD values are presented in each bar. *, $P < 0.05$; **, $P < 0.01$; and $^{\#}$, $P < 0.001$ versus DMSO treatment.

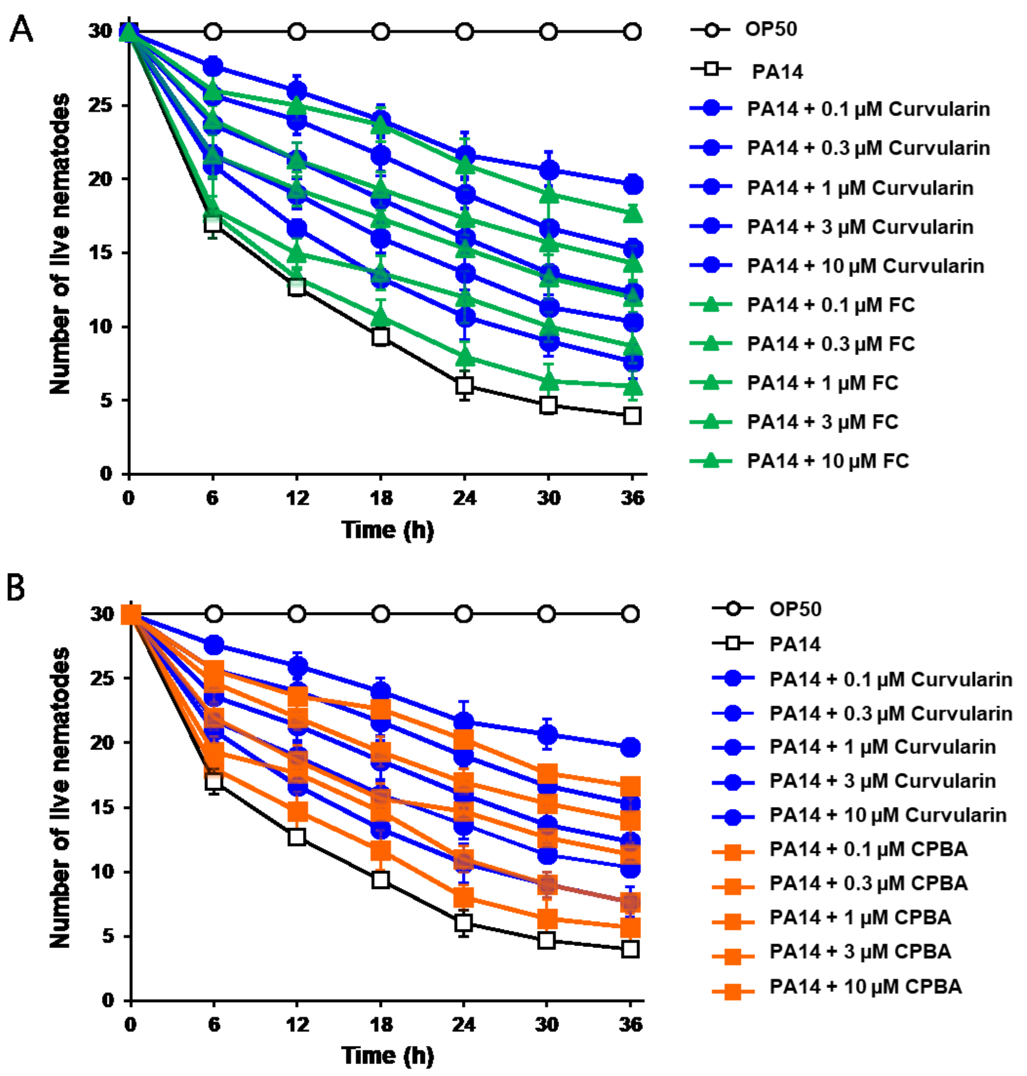

Supplementary Figure 17. Comparison of *in vivo* antivirulence activity of curvularin with known QS antagonists. Antivirulence effects of curvularin in a *C. elegans* infection model. Thirty worms were introduced on lawns of of *E. coli* OP50 (open circles) or PA41 (filled circles) on plates in the presence of different concentrations of curvularin, FC (**A**), or CPBA (**B**). Live nematodes were counted every 5 h for 30 h. Furanone C-30 (FC) and N-cyclopentylbutyramide (CPBA) are known antagonists of LasR and RhlR, respectively. Two independent experiments were performed in triplicate, and the mean ± SD values are displayed in each graph.
